# Targeted Microbial Shifts and Metabolite Profiles Were Associated with Clinical Response to an Anti-Inflammatory Diet in Osteoarthritis

**DOI:** 10.3390/nu17172729

**Published:** 2025-08-22

**Authors:** Marta Sala-Climent, Kevin Bu, Roxana Coras, Martha Cedeno, Simone Zuffa, Jessica Murillo-Saich, Helena Mannochio-Russo, Celeste Allaband, Michal K. Hose, Anna Quan, Soo-In Choi, Katherine Nguyen, Shahrokh Golshan, Rebecca B. Blank, Tiffany Holt, Nancy E. Lane, Rob Knight, Jose Scher, Pieter Dorrestein, Jose Clemente, Monica Guma

**Affiliations:** 1 Department of Medicine, School of Medicine, University of California San Diego, San Diego, CA 92093, USA; msalacliment@health.ucsd.edu (M.S.-C.); roxana.juverdeanu@gmail.com (R.C.); marthacedeno8313@gmail.com (M.C.); jdmurillosaich@health.ucsd.edu (J.M.-S.); s6choi@health.ucsd.edu (S.-I.C.); khn022@health.ucsd.edu (K.N.); 2 Department of Genetics and Genomic Sciences, Icahn School of Medicine at Mount Sinai, New York, NY 10029, USA; kbu314@gmail.com; 3 Skaggs School of Pharmacy and Pharmaceutical Sciences, University of California San Diego, San Diego, CA 92093, USA; szuffa@health.ucsd.edu (S.Z.); hmannochiorusso@health.ucsd.edu (H.M.-R.); pdorrestein@health.ucsd.edu (P.D.); 4 Department of Pediatrics, School of Medicine, University of California San Diego, San Diego, CA 92093, USA; callaban@health.ucsd.edu (C.A.); rkinght@health.ucsd.edu (R.K.); 5 VA San Diego Healthcare System, San Diego, CA 92161, USA; michalkalli.hose@va.gov (M.K.H.); anna.quan@va.gov (A.Q.); 6 Department of Psychiatry, School of Medicine, University of California San Diego, San Diego, CA 92093, USA; sgolshan@health.ucsd.edu (S.G.); tholt@health.ucsd.edu (T.H.); 7 Medicine and Rheumatology, NYU Grossman School of Medicine, New York, NY 10016, USA; rebecca.blank@nyulangone.org (R.B.B.); jose.scher@nyulangone.org (J.S.); 8 Medicine and Rheumatology, UC Davis Health, Sacramento, CA 95817, USA; nelane@ucdavis.edu; 9 Center for Microbiome Innovation, University of California San Diego, San Diego, CA 92093, USA; 10 Department of Computer Science and Engineering, University of California San Diego, San Diego, CA 92093, USA; 11 Department of Bioengineering, University of California San Diego, San Diego, CA 92093, USA

**Keywords:** nutrition intervention, osteoarthritis, anti-inflammatory diet, microbiome, metabolome, microbiome–metabolome interactions

## Abstract

**Introduction:** Osteoarthritis (OA) is a chronic degenerative joint disease with limited treatment options focused primarily on symptom management. Emerging evidence suggests that dietary interventions may influence inflammation and pain through modulation of the gut microbiome and metabolome. **Methods:** We conducted a 4-week open-label pilot trial evaluating the effects of an anti-inflammatory dietary intervention (ITIS diet) in 20 patients with knee OA (ClinicalTrials.gov ID: NCT05559463, registered prior to enrollment; sponsor: University of California, San Diego; responsible party: Monica Guma; study start date: 1 October 2021). The following were assessed before and after the intervention: (1) clinical outcomes; (2) gut and salivary microbiomes; and (3) salivary, stool, and plasma metabolomes. Responders were defined as patients achieving ≥30% reduction in Western Ontario and McMaster Universities Arthritis Index (WOMAC) pain scores. **Results:** The ITIS diet was well-tolerated, with good adherence (66.2%) and a significant improvement in clinical outcomes, including reduced pain and improved overall health measured with the visual analog scale (VAS). Responders (*n* = 8) showed distinct gut microbiome and metabolome profiles compared to non-responders (*n* = 12). Notably, taxa within the *Lachnospiraceae* family exhibited dynamic, bidirectional shifts post-intervention: *Anaerostipes* and *Limivivens* were enriched among responders and negatively correlated with pain scores, while *Oliverpabstia* and *Fusicatenibacter* were depleted following dietary intervention. These taxa also showed strong correlations with anti-inflammatory metabolites, including hydroxydecanoic acid derivatives and pyridoxine. Furthermore, subsequent network analysis revealed more structured and selective microbiome–metabolome interactions in responders, specifically post-intervention. **Conclusions:** This pilot study shows that a short-term anti-inflammatory dietary intervention was associated with meaningful changes in the gut microbiome and metabolome. Members of the Lachnospiraceae family emerged as key taxa associated with pain reduction and anti-inflammatory metabolite production. Our findings suggest that specific microbial responses—rather than global diversity changes—may underlie dietary responsiveness in OA. Although exploratory and limited by sample size, our results support further investigation into personalized, microbiome-informed nutritional strategies for OA management.

## 1. Introduction

Osteoarthritis (OA) is the most common joint disease worldwide, affecting 10% of men and 18% of women over the age of 60 [1]. Clinical manifestations include pain, morning stiffness, and crepitus with joint movement, all of which negatively affect quality of life. Current management strategies for OA primarily focus on symptom relief. Many patients also seek non-pharmacological approaches—such as dietary changes and exercise—in addition to pharmacological treatments.

Diet has been linked to both disease onset and symptomatology in various rheumatic conditions. For instance, adherence to a Western dietary pattern has been associated with an increased risk of knee OA, whereas a prudent diet rich in fruits and vegetables has been correlated with a reduced risk [2]. Several components of Western diets, including refined sugars, gluten [3], trans- and saturated fatty acids (FA) [4], dairy products [5], and red meat [6], have been shown to promote inflammation. Conversely, Western dietary patterns are often low in essential nutrients—such as long-chain omega-3 polyunsaturated FA (PUFA) [7], monounsaturated FA (MUFA) [8], antioxidants [9], phytochemicals [10], and flavonoids [11]—that are known to support overall health and reduce inflammation [12].

Diet can influence inflammation by modulating the gut microbial ecology, which is highly dynamic and rapidly responsive to dietary changes [13]. The gut microbiota plays a crucial role not only in absorbing essential vitamins, but also in shaping metabolic responses to nutrients and regulating pro- and anti-inflammatory mediators [14]. For example, certain bacterial species, such as *Akkermansia muciniphila*, have been linked to maintaining intestinal barrier integrity and promoting anti-inflammatory effects through the production of short-chain fatty acids (SCFAs) and modulation of host metabolic pathways [15].

Although studies have shown an association between poor dietary quality and increased inflammation and pain in patients with OA, there is still a relative scarcity of research specifically focused on the role of diet in OA [16,17,18]. Some studies have explored the relationship between nutrition and OA outcomes using food frequency questionnaires.

One study found that higher adherence to a Mediterranean diet (MD) was associated with improved quality of life, reduced pain and disability, and fewer depressive symptoms in knee OA subjects [19]. Another reported that greater adherence to an MD was linked to a lower prevalence of knee OA, even after adjusting for potential confounding factors such as age, race, BMI, and sex [20]. A longitudinal cohort study also found that greater adherence to an MD was associated with a reduced risk of pain worsening and symptomatic knee OA over four years. However, no significant association was observed with the incidence of radiographic knee OA [21].

Only one clinical trial has investigated whether a Mediterranean-like diet could reduce inflammation and cartilage degradation in patients with OA. The study found that while the diet was associated with a reduction in the pro-inflammatory cytokine IL-1α and showed a trend toward decreased cartilage degradation, the overall biomarker response was limited. These findings highlight the need for further research [22].

While multiple studies have investigated the association between the microbiome, the metabolome, and diet interventions in healthy controls and other diseases [23,24], there is still little data on the effect of dietary intervention on the microbiome, circulating metabolites, and clinical response in OA patients [25,26].

To assess the potential impact of dietary intervention in OA, we first adapted the previously employed MD and determined ways to make it more suitable for OA patients. Importantly, the MD does not include a variety of ingredients and compounds known to have anti-inflammatory properties. For instance, fruits with enzymatic proteins, such as papain and bromelain (papaya, mango, pineapple) [27], PUFA and MUFA [9,28,29], ginger [30,31], turmeric [32], green tea [33], miso [34], and chia seeds [35], are not part of the MD. In addition, some vegetables included in the MD—such as tomatoes, eggplants, and potatoes—contain solanine, a glycoalkaloid that increases intestinal permeability and has been suggested to be detrimental in arthritogenic pathologies [36].

To address these limitations, we developed the ITIS diet—an anti-inflammatory dietary intervention previously shown to exert beneficial effects in patients with rheumatoid arthritis (RA) [37]. It is an omnivorous diet based on the MD, but with several modifications. Combining multiple strategies to decrease inflammation may represent a more effective approach for those suffering from painful OA.

This manuscript describes data from an open-label pilot trial conducted to evaluate clinical and biological outcomes of a 4-week ITIS diet intervention in OA patients. We report changes in pain score as the primary clinical outcome, with metabolomic and microbiome profiles before and after the intervention as secondary outcomes. Finally, we examine the relationship between metabolomic and microbial changes as potential mediators of clinical outcomes.

## 2. Materials and Methods

### 2.1. Study Participants and Study Design

A prospective, open-label pilot trial was conducted to evaluate the clinical and biological outcomes of a 4-week isocaloric ITIS diet. We recruited patients with a diagnosis of knee OA with a visual analog pain knee score of between 20 and 80 during the last 7 days, and without changes in therapy during the previous 3 months. The study was approved by the Institutional Board Review of University of California San Diego (#161474) on 26 January 2022, and all patients signed an informed consent form. Details on enrollment, dietary recommendations, provided food items, and diet score calculations are presented in Supplementary Tables S1–S4. A STROBE-compliant flow diagram illustrating patient enrollment and follow-up is provided in Supplementary Figure S1.

**Figure 1:**
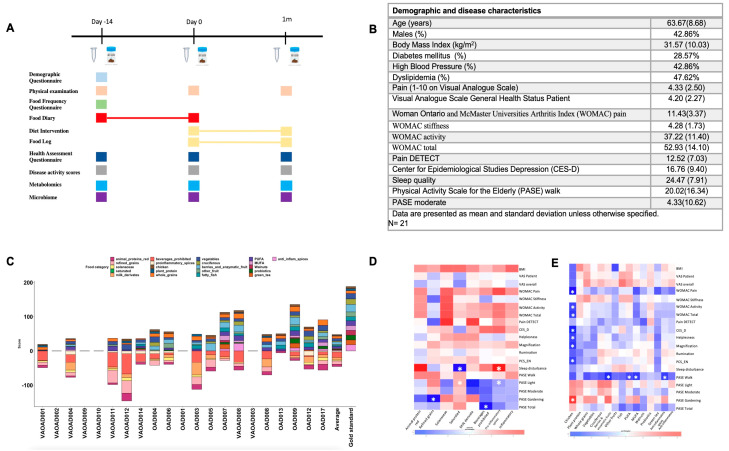
Trial design and baseline characteristics of patients, clinical and diet scores. (**A**) Trial design; (**B**) demographic and clinical characteristics of patients at recruitment; (**C**) bar plot representing the diet scores for each patient at day 0; the last bar shows the gold standard score according to the proposed anti-inflammatory diet; negative values correspond to consumption of pro-inflammatory foods, prohibited in the proposed diet; patients with missing bar plots did not fill the diet log; (**D**) heatmap representing correlation (Spearman) between pro-inflammatory food groups (columns) and clinical scores (rows). The significant correlations (*p* < 0.05) are signaled with a white asterisk; (**E**) heatmap representing correlation (Spearman) between anti-inflammatory food groups (columns) and clinical scores (rows). Significant correlations (*p* < 0.05) are signaled with a white asterisk. WOMAC—Woman Ontario and McMaster Universities Arthritis Index, CES-D—Center for Epidemiological Studies Depression, PCS-EN—Pain Catastrophizing Scale in English, VAS MD—visual analog scale general health status physician; PUFA—polyunsaturated fatty acids; MUFA—monounsaturated fatty acids.

The study was performed from September 2021 to March 2023 at the University of California San Diego. The trial was registered on ClinicalTrials.gov (ID: NCT05559463) prior to the enrollment of the first participant (sponsor: University of California, San Diego; responsible party: Monica Guma; study start date: 1 October 2021). Research visits were performed on 3 occasions during the study (Figure 1A). During their first visit (D-14), we conducted initial assessments, collected clinical and biological data, and instructed patients to maintain their usual diet while recording their daily intake in a diet log for the following two weeks. On their second visit (D0), two weeks later, patients were given instructions on how to follow the energy-adjusted ITIS diet and were also asked to complete a daily diet log for 2 weeks after starting the diet. On their third visit (1 m), we evaluated diet adherence, trial satisfaction, and clinical parameters. The measurements taken on day -14 and day 0 were utilized to establish the baseline characteristics of the patients, as no statistically significant differences were observed between these timepoints (Supplementary Table S5). For all subsequent analyses, the mean values of measurements from day -14 and day 0 were calculated and employed to represent the baseline data. The dates were defined in reference to the start of the diet (day 0). Pain and overall health were self-evaluated by the patient, using a visual analog scale (VAS) that ranged from 0 to 10. Additionally, Western Ontario and McMaster Universities Arthritis Index (WOMAC), Pain Catastrophizing Scale (PCS), PROMIS sleep disturbance, Physical Activity Scale for the Elderly (PASE), Center for Epidemiological Studies Depression (CES-D), and painDETECT questionnaires were collected in each of the three visits. Blood samples were also collected at each visit by research personnel into 10 mL BD Vacutainer blood collection tubes containing EDTA. The tubes were centrifuged for 20 min at 2000× rpm, and plasma were transferred into 1.7 mL tubes and immediately frozen and stored at −80 °C until analysis. Patients were asked to collect stool and saliva samples at home after each visit in special sealed plastic containers provided on the day of each visit. Stool samples were collected using nylon-flocked swabs and immediately placed into 1 mL matrix tubes (Thermo Fisher Scientific, Waltham, MA, USA, catalog #3741) containing 400 µL of 95% ethanol. DNA was subsequently extracted following standard ethanol-based protocols. This method was specifically designed to minimize well-to-well contamination and allow for simultaneous microbiome and metabolite analyses. Saliva samples were collected in tubes that were also prefilled with 400 µL of 95% ethanol. All stool and saliva samples were shipped in temperature-controlled containers, aliquoted upon arrival, and stored at −80 °C until further processing. The sample size was based on feasibility and typical sample sizes for pilot studies of this type. No formal power calculation was conducted. We aimed to enroll 20–25 participants to assess feasibility and generate preliminary data on dietary response in OA patients. The final number of completers (*n* = 21) aligns with standard sample sizes in exploratory pilot trials.

### 2.2. Primary and Secondary Outcomes

Change in pain score was the primary outcome. In accordance with Conaghan et al., we considered a 30% reduction in WOMAC pain to be an appropriate threshold for patients with OA [38]. Secondary outcomes included changes in other clinical scores such as VAS, activity or sleep quality, gut and saliva microbiota, and gut and plasma metabolome.

### 2.3. Metabolome Sample Preparation for LC-MS/MS Analyses

For blood plasma and serum, 50 µL samples were processed using a Phree kit for phospholipid removal, followed by 100% methanol extraction, centrifugation, and drying before storage at −80 °C. Saliva samples were mixed with 100% ethanol, sonicated, incubated at −20 °C for protein precipitation, centrifuged, and the supernatant was dried and stored. Stool swabs were incubated overnight in ethanol/water, sonicated, centrifuged, and the supernatant was collected, dried, and stored. Before LC-MS/MS analysis, all samples were resuspended in 50:50 methanol/water containing 2 µM sulfadimethoxine as an internal standard. Expanded methodology, including detailed sample extraction steps, instrument parameters, and data acquisition and processing pipelines (using a Vanquish UPLC coupled to a Q Exactive Orbitrap MS, MZmine2 processing, and GNPS molecular networking), can be found in the Supplementary Materials.

### 2.4. Microbiome Data Acquisition

The UC San Diego Microbiome Core performed nucleic acid extractions utilizing previously published protocols [39]. Briefly, samples were purified using the MagMAX Microbiome Ultra Nucleic Acid Isolation Kit (Thermo Fisher Scientific) and automated on KingFisher Flex robots (Thermo Fisher Scientific). DNA was quantified using a PicoGreen fluorescence assay (Thermo Fisher Scientific). Bacterial 16S rRNA gene amplicon sequencing (V4 region, 150 bp read length, paired-end) was performed on the Illumina MiSeq.

### 2.5. Microbiome Data Processing

After 16S rRNA sequencing, microbiome data was analyzed using QIIME 2 v-2020.8.0. Sequences were demultiplexed and denoised with DADA2 and taxonomies classified using GreenGenes2. Alpha diversity (Shannon Index) and beta diversity (Unweighted UniFrac) were estimated using rarefaction at 10,000 reads. Feature tables were normalized to obtain relative abundances for each taxa on a per-sample basis.

### 2.6. Statistical Analyses

Continuous variables are presented as the mean ± standard deviation (SD), whereas categorical variables are summarized as the number (percentage) of subjects. Nonparametric tests were used to compare means across 2 groups, and the *p* values obtained were adjusted for multiple testing using the Benjamini–Hochberg procedure where necessary. As various patients were missing values for a particular datatype (with only 12 complete cases across all datatypes), each plot shows the number of samples contributing to the analyses within that datatype. Metabolomic data were first normalized via total-sum scaling, mean-centering, and log-transformation, while microbiome data was normalized via total-sum scaling (without log-transform due to the small sample size and sparsity of the data). The advantages and disadvantages of normalization techniques (such as rarefaction, total-sum scaling) and transformations (e.g., centered log-ratio) used to address compositional data have been thoroughly described [40,41,42]. Compositionality in particular is known to induce spurious associations; however, the small sample size of our study precludes the usage of techniques such as ANCOM-BC due to the assumptions of sufficient *n* for convergence theorems to hold [43]. The robust-centered log ratio (rCLR) transformation is also not well-suited to this setting due to the high sparsity of the data. Specifically, rCLR performed on microbiome data causes non-zero values to become negative, while 0’s remain 0; this paradoxically causes an apparent ‘increase’ in value when the taxa present in a pre-diet sample becomes absent in the post-sample. Moreover, we choose to perform univariate testing over inference on contrasts and ratios due to ease of interpretability [44]. Finally, given that recent studies suggest that the relative sensitivity and specificity of tools is both comparable and dependent upon sample type [45], non-parametric tests were used to account for the low sample size and non-normality of the data in favor of interpretability of features. Differences in alpha diversity (as measured by Shannon Index) between responders and non-responders were assessed using the Wilcoxon rank-sum test, and differences in alpha diversity between pre- and post- intervention timepoints were tested using the Wilcoxon signed-rank test. Beta diversity (unweighted UniFrac) was compared using PERMANOVA (with 1000 iterations) and visualized using PCoA. Univariate statistical testing for individual features was performed using Wilcoxon rank-sum or Wilcoxon signed-rank non-parametric tests. Due to the limitations of the sample size, *p*-values were adjusted using FDR-BH, but both corrected and noncorrected *p*-values were reported in differential analyses plots. Features were ranked based on nonparametric *p*-values and reported with FDR-corrected levels of significance as well as Cohen’s D effect sizes [46]. Bars were then colored by the direction of enrichment (e.g., pre- or post-dietary-intervention) along with the threshold of significance. “PreFDR” indicates significant prior to correction (*p* < 0.05), post-FDR indicates after correction (q < 0.05), and NS indicates *p* > 0.05. Correlation analyses were conducted using Spearman correlation (or Pearson when distributional assumptions were met), and confounders and covariates were controlled for using generalized linear models. All statistical tests were performed in Python 3.7.3 using the scipy.stats library and in R 4.2.1 using the base library. Normalized CSV files (microbiome, metabolome, and metadata) were generated using the rCLR transformation and uploaded to the OmicsAnalyst 2.042 platform for multi-omics integration using DIABLO (Data Integration Analysis for Biomarker Discovery Using Latent Components) [47]. DIABLO is a supervised multivariate method that identifies correlated features across omics layers while optimizing the discrimination between predefined groups. In our analysis, samples were grouped according to their timepoint (baseline or post-diet), and no paired sample design was used. Model performance was evaluated using 5-fold cross-validation, repeated 10 times, with accuracy and balanced error rate (BER) as performance metrics. To visualize associations between microbiome and metabolome features, we calculated pairwise Pearson correlations between the top discriminant features selected by DIABLO within each group (baseline and post-diet and responders and non-responders). Only statistically significant correlations (|r| > 0.7, *p* < 0.05) were retained. To enhance interpretability and reduce visual complexity, the maximum number of edges per network was limited to 200. The bar plots were created using the *ggboxplot* function and the line plots using the *ggline* function in the *ggupbr* package. The heatmaps were built using the heatmap.2 function, based on the Spearman correlation coefficients and corresponding *p* values.

## 3. Results

### 3.1. Patient Demographics, Disease Characteristics and Diet at Baseline

Twenty-one OA patients completed the ITIS diet trial, as outlined in Figure 1A. Demographics and disease characteristics are summarized in Figure 1B. The average age was 63.67 ± 8.68 years, and 42.86% were male. The mean body mass index (BMI) was 31.57 ± 10.03. Comorbidities included diabetes mellitus in 28.57% of patients, hypertension in 42.86%, and dyslipidemia in 47.62%.

At baseline, pain was assessed using the WOMAC pain scale (11.18 ± 2.78) and the patient-reported VAS (4.43 ± 2.06). The total WOMAC score was 52.61 ± 13.97. Diet score, calculated from a 2-week daily diet log prior to the intervention, was used to characterize the baseline diet. The average total diet score was 32.82 ± 28.65, with a mean pro-inflammatory score of −29.71 ± 26.58 and a mean anti-inflammatory score of 62.53 ± 34.08 (Supplementary Table S6).

The most consumed pro-inflammatory ingredients were animal protein (red meat, beef, and burgers), forbidden beverages (alcohol, soda, coffee), and refined grains (white processed bread, wheat tortillas, crackers, and cookies). In contrast, patients reported good intake of vegetables, berries, and enzymatic fruits, but low consumption of probiotics, PUFA, MUFA, and anti-inflammatory spices (Figure 1C, Supplementary Table S6).

We next examined correlations between pro-inflammatory foods (Figure 1D), anti-inflammatory foods (Figure 1E), and recorded outcomes. Notably, chicken consumption showed a negative correlation with seven outcomes: WOMAC pain, WOMAC activity, WOMAC total score, CES-D, helplessness, magnification, and PCS.

### 3.2. Effect of the ITIS Diet on Clinical Outcome

**Figure 2:**
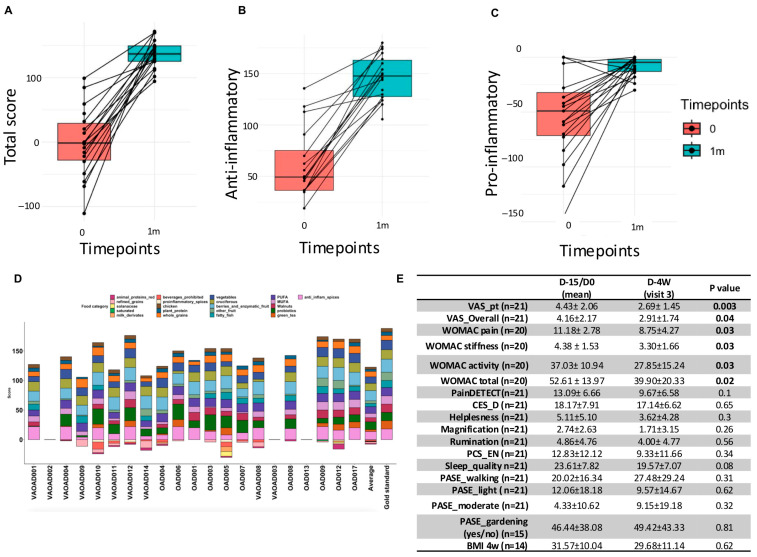
Adherence to diet and improvement in clinical scores after diet. (**A**) Improvement in total score after diet; (**B**) improvement in anti-inflammatory score after diet; (**C**) improvement in pro-inflammatory score after diet; (**D**) bar plot representing the diet scores for each patient after the intervention; the last bar shows the gold standard score according to the proposed anti-inflammatory diet; negative values correspond to consumption of pro-inflammatory foods, prohibited in the proposed diet; (**E**) table of difference between baseline and after diet in all the outcomes measured.

The dietary intervention was well-received and well-tolerated, with no adverse events reported. Adherence to the intervention was reflected in a significant increase in the diet score (maximum: 212), which rose from 32.82 ± 28.65 at day 0 to 140.35 ± 46.71 at 1 m, corresponding to a 66.2% adherence rate (*p* < 0.001) (Figure 2A, Supplementary Table S6). The total anti-inflammatory score improved following the intervention (Figure 2B), while the pro-inflammatory score showed a corresponding decrease (Figure 2C). Adherence was assessed individually, revealing that dietary recommendations were consistently followed. By the end of the trial, participants had achieved at least 66.2% of the total diet score (Figure 2D). Clinical outcomes improved post-intervention (Figure 2E). Specifically, patient-reported VAS pain decreased from 4.43 ± 2.06 at baseline to 2.69 ± 1.45 post-intervention (*p* = 0.003), while VAS overall health improved from 4.16 ± 2.17 to 2.91 ± 1.74 (*p* = 0.04). WOMAC pain decreased from 11.18 ± 2.78 to 8.75 ± 4.27 (*p* < 0.03), and WOMAC total score improved from 52.61 ± 13.97 to 39.90 ± 20.33 (*p* = 0.02).

### 3.3. Effects of the ITIS Diet on Microbiome and Metabolome

**Figure 3:**
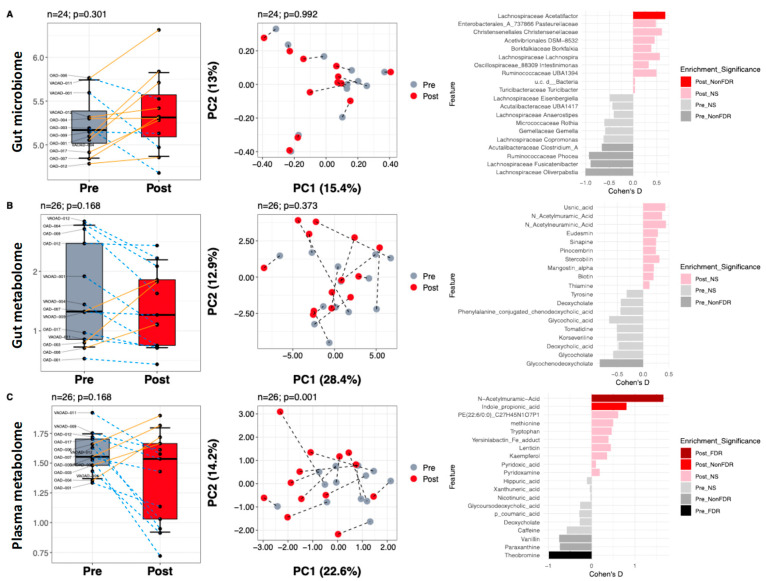
Impact of diet on gut microbiome, gut metabolome, and plasma metabolome irrespective of responder status. Alpha diversity, beta diversity, and differential abundance analyses for the gut microbiome (**A**), gut metabolome (**B**), and plasma metabolome (**C**). In the leftmost column, paired Wilcoxon signed-rank tests were conducted for differences in alpha diversity, and PERMANOVA was used for assessing differences in composition in the center column. In the rightmost column, paired Wilcoxon signed-rank tests identified differential features. *Post_FDR* indicates enrichment of a feature in post-intervention samples with q < 0.05 after false discovery rate (FDR) correction; *Pre_FDR* indicates enrichment in pre-intervention samples with q < 0.05 after FDR correction. *Post_NonFDR* indicates enrichment (*p* < 0.05) of a feature in post-intervention samples not corrected by FDR. *Pre_NonFDR* indicates enrichment (*p* < 0.05) in pre-intervention samples not corrected by FDR. NS indicates *p* > 0.05.

We first evaluated the effect of the ITIS diet on the microbiome and metabolome across all patients. The relative abundance of microbes in stool and saliva samples before and after the intervention is shown in Supplementary Figure S2. Figure 3A–C presents alpha diversity, beta diversity, and top differential features for each data type (gut microbiome, gut metabolome, and plasma metabolome).

In the gut microbiome, diversity did not differ between baseline and post-intervention (alpha diversity: *p* = 0.301, Wilcoxon signed-rank; beta diversity: *p* = 0.992, PERMANOVA), although there was a trend toward increased alpha diversity (Figure 3A, left). Taxonomic analysis revealed a significant enrichment in *Acetatifactor* and a depletion of *Clostridium*, *Phocea*, *Fusicatenibacter*, and *Oliverpabstia* after the dietary intervention (Figure 3A, right).

In the gut metabolome (Figure 3B), alpha diversity showed a non-significant decrease after the intervention (*p* = 0.168, Wilcoxon signed-rank), and beta diversity was also not different (*p* = 0.373, PERMANOVA). However, we observed a decrease in glycone-dihydroxylated bile acid abundance (Wilcoxon signed-rank *p* < 0.05) (Figure 3B, right).

In the plasma metabolome (Figure 3C), alpha diversity showed a non-significant change (*p* = 0.168, Wilcoxon signed-rank), but beta diversity differed between baseline and post-intervention (*p* = 0.001, PERMANOVA). We identified an enrichment in compounds annotated as N-acetylmuramic acid, along with a decrease in vanillin, paraxanthine, and theobromine (Wilcoxon signed-rank q < 0.05) (Figure 3C, right).

### 3.4. Baseline Diet Scores After ITIS Diet Intervention Are Associated with Clinical Outcome Response to the ITIS Diet

**Figure 4:**
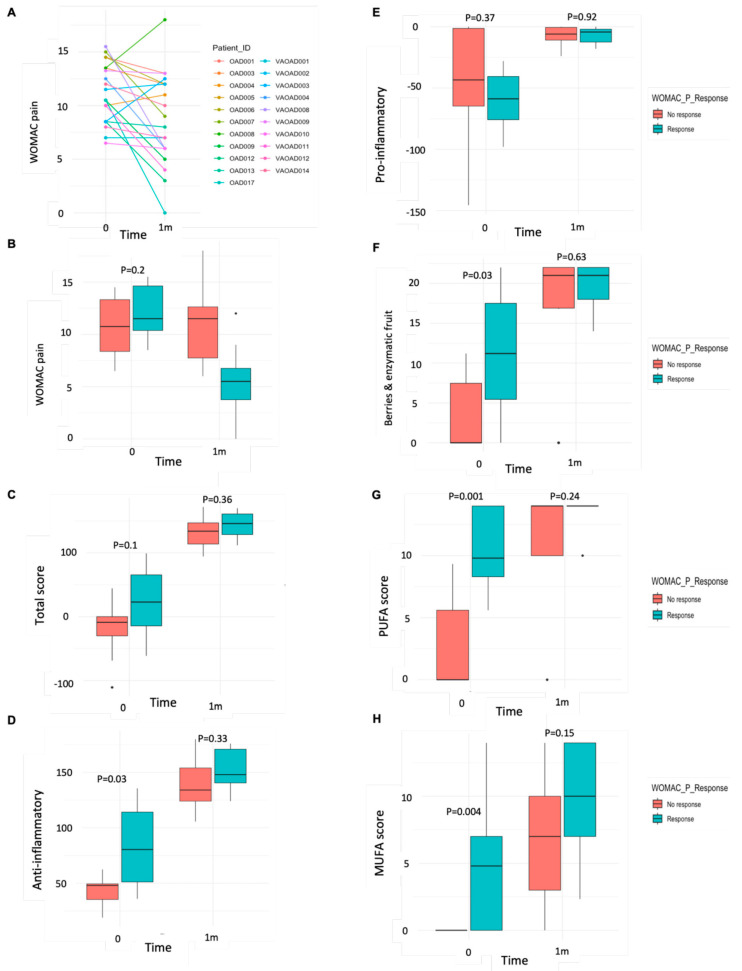
Dietary-related changes by response at baseline and after diet. (**A**) Evolution of WOMAC pain score in each patient; (**B**) box plot divided by patients who will present an improvement of 30% of WOMAC pain (response—blue), compared to patients who will have a lower improvement in pain (non-response—red); (**C**) box plot of total dietary score variation before and after the intervention by response; (**D**) anti-inflammatory score at baseline and after diet by response; (**E**) pro-inflammatory score at baseline and after diet by response; (**F**) berries and enzymatic fruit score consumption over time by response; (**G**) polyunsaturated fatty acid (PUFA) score before and after diet by response; (**H**) monounsaturated fatty acid (MUFA) score before and after dietary intervention by response. A paired Wilcoxon test was used to assess differences in the intake of foods before and after the intervention.

To evaluate the efficacy of the ITIS diet, we categorized patients as responders (R) or non-responders (NR) using a clinically meaningful threshold of a ≥30% improvement in WOMAC pain. This classification resulted in 8 R and 12 NR. Figure 4A shows the progression of WOMAC pain for each patient, and Figure 4B compares changes in WOMAC pain between groups. At baseline, WOMAC pain scores did not differ between R and NR (R: 12.13 ± 2.63 vs. NR: 10.56 ± 2.82; *p* = 0.20) (Supplementary Table S7).

Total diet scores before and after the intervention were also similar between groups [baseline: R: 22.01 ± 58.62 vs. NR: −19.51 ± 46.68; *p* = 0.10; post-diet: R: 144.63 ± 21.18 vs. NR: 131.15 ± 24.68; *p* = 0.36] (Figure 4C; Supplementary Tables S8 and S9). However, when the diet score was separated into pro- and anti-inflammatory indices, R had a higher anti-inflammatory score at baseline (R: 88.99 ± 38.14 vs. NR: 42.71 ± 14.00; *p* = 0.03), with no difference in the pro-inflammatory score (*p* = 0.37) (Figure 4D,E; Supplementary Tables S8 and S9).

Further analysis of individual anti-inflammatory components revealed that R consumed more berries and enzymatic fruits (*p* = 0.03), PUFAs (*p* = 0.001), and MUFAs (*p* = 0.004) at baseline compared to NR (Figure 4F–H; Supplementary Table S9). Additional differences included higher scores for whole grains (*p* = 0.03), vegetables (*p* = 0.06), other fruits (*p* = 0.004), probiotics (*p* = 0.01), anti-inflammatory spices (*p* = 0.03), and cruciferous vegetables (*p* = 0.01) (Supplementary Figure S3A–F). Post-intervention, these differences were no longer significant, except for vegetable intake, which remained higher in R (*p* = 0.07) (Supplementary Figure S3C; Supplementary Table S9).

### 3.5. Baseline Specific Taxa and Metabolites Were Not Associated with Clinical Outcome Response to the ITIS Diet

**Figure 5:**
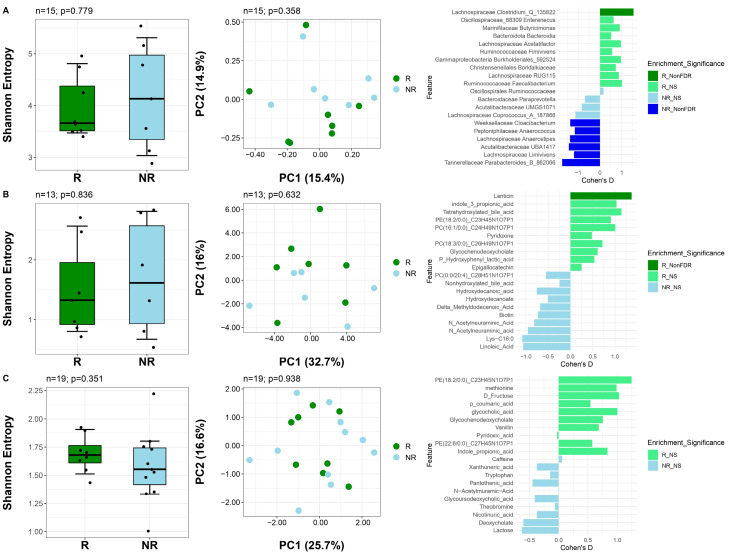
Association of baseline gut microbiome, gut metabolome, and plasma metabolome features with responder status. Alpha diversity, beta diversity, and differential abundance analyses for the gut microbiome (**A**), gut metabolome (**B**), and plasma metabolome (**C**) at baseline comparing responders and non-responders. In the leftmost column, Wilcoxon rank-sum tests were conducted for differences in alpha diversity, and PERMANOVA was used for assessing differences in composition in the center column. In the rightmost column, Wilcoxon rank-sum tests identified differential features. *R_FDR* indicates enrichment of a feature in responders with q < 0.05 after FDR correction; *NR_FDR* indicates enrichment of a feature in non-responders with q < 0.05 after FDR correction. *R_NonFDR* indicates enrichment (*p* < 0.05) of a feature in responders not corrected by FDR. *NR_NonFDR* indicates enrichment (*p* < 0.05) in non-responders not corrected by FDR. NS indicates *p* > 0.05.

We next assessed whether participants’ baseline microbial and metabolomic features were associated with response status or degree of response. Overall, diversity measures at baseline were not linked to response. However, specific taxa and metabolites differed between groups (Figure 5).

In the gut microbiome (Figure 5A), alpha diversity did not differ between R and NR (*p* = 0.779, Wilcoxon rank-sum), and neither did beta diversity (*p* = 0.358, PERMANOVA). Nonetheless, R showed an enrichment of *Clostridium* and a depletion of *Anaerococcus*, *Cloacibacterium* (Weeksellaceae), *Anaerostipes*, *Limivivens*, and *Parabacteroides* (all Wilcoxon signed-rank *p* < 0.05).

In the gut metabolome (Figure 5B), diversity metrics were also similar between groups (alpha diversity: *p* = 0.836; beta diversity: *p* = 0.632). The only metabolite enriched in R was lenticin (*p* < 0.05).

In the plasma metabolome (Figure 5C), no significant differences were observed (alpha diversity: *p* = 0.351; beta diversity: *p* = 0.938), and no individual metabolites differed between groups. However, NR displayed greater variance in alpha diversity.

Together, these findings indicate that differences between R and NR after the dietary intervention are not explained by distinct baseline microbiome or metabolome diversity profiles. Post-intervention analyses revealed only a few microbial and metabolic differences between groups (Supplementary Figure S4).

### 3.6. Baseline-to-Post Diet Changes in the Gut Microbiome and Plasma Metabolome Differ by Clinical Response to the ITIS Diet

We next investigated changes in the gut microbiome, gut metabolome, plasma metabolome, salivary microbiome, and salivary metabolome in patients who responded to the dietary intervention.

**Figure 6:**
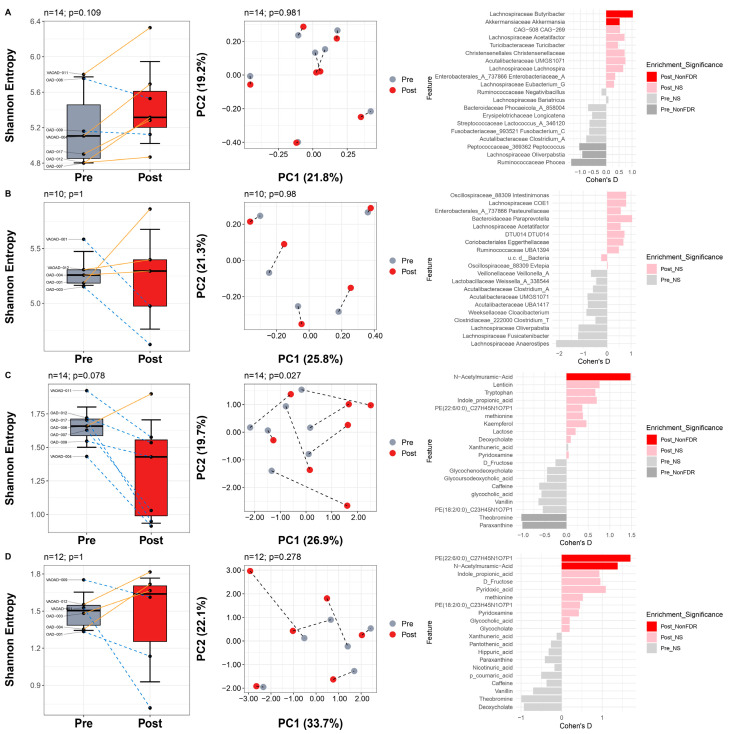
Changes in gut microbiome and plasma metabolome profile from baseline to after diet separated by responders and non-responders. Alpha diversity, beta diversity, and differential abundance analyses for the gut microbiome in responders (**A**), gut microbiome in non-responders (**B**), plasma metabolome in responders (**C**), and plasma metabolome in non-responders (**D**) comparing pre- and post-dietary intervention values. In the leftmost column, paired Wilcoxon signed-rank tests were conducted for differences in alpha diversity, and PERMANOVA was used for assessing differences in composition in the center column. In the rightmost column, paired Wilcoxon signed-rank tests identified differential features. *Post_FDR* indicates enrichment of a feature in post-intervention samples with q < 0.05 after false discovery rate (FDR) correction; *Pre_FDR* indicates enrichment in pre-intervention samples with q < 0.05 after FDR correction. *Post_NonFDR* indicates enrichment (*p* < 0.05) of a feature in post-intervention samples not corrected by FDR. *Pre_NonFDR* indicates enrichment (*p* < 0.05) in pre-intervention samples not corrected by FDR. NS indicates *p* > 0.05.

In R, the gut microbiome showed a trend toward increased alpha diversity (*p* = 0.109, Wilcoxon signed-rank) but no significant shift in beta diversity (*p* = 0.981, PERMANOVA) (Figure 6A). Nonetheless, we observed an enrichment of *Butyricibacter* and *Akkermansia*, and a depletion of *Oliverpabstia* and *Phocea* (all *p* < 0.05), suggesting potential microbiome shifts associated with response. In contrast, NR exhibited minimal changes, with no differences in alpha diversity (*p* = 1) or beta diversity (*p* = 0.980) (Figure 6B), and no differential microbial features.

In the plasma metabolome, R displayed distinct pre- and post-diet differences, with trends toward changes in alpha diversity (*p* = 0.078) and significant changes in beta diversity (*p* = 0.027) (Figure 6C). At the feature level, N-acetylmuramic acid was enriched, while theobromine and paraxanthine were depleted (all *p* < 0.05). In NR, plasma metabolome changes were minimal, with no differences in alpha diversity (*p* = 1) and no significant shift in beta diversity (*p* = 0.278) (Figure 6D). Only two metabolites were enriched post-diet, one of which was N-acetylmuramic acid.

### 3.7. Beyond Individual Changes: Differential Microbiome and Metabolome Shifts in Responders vs. Non-Responders to the ITIS Diet Reveal Distinct Dietary Effects

To better understand the differential impact of the dietary intervention, we examined whether changes in the gut microbiome, gut metabolome, and plasma metabolome were associated with the degree of clinical response. Unlike previous analyses that focused on within-group pre–post changes, this approach directly compared the magnitude and direction of these changes between R and NR.

For each dataset, we calculated the difference (delta) between pre- and post-intervention relative abundance values within R and NR separately. We then compared these deltas between groups, identifying features where the degree of change differed.

**Figure 7:**
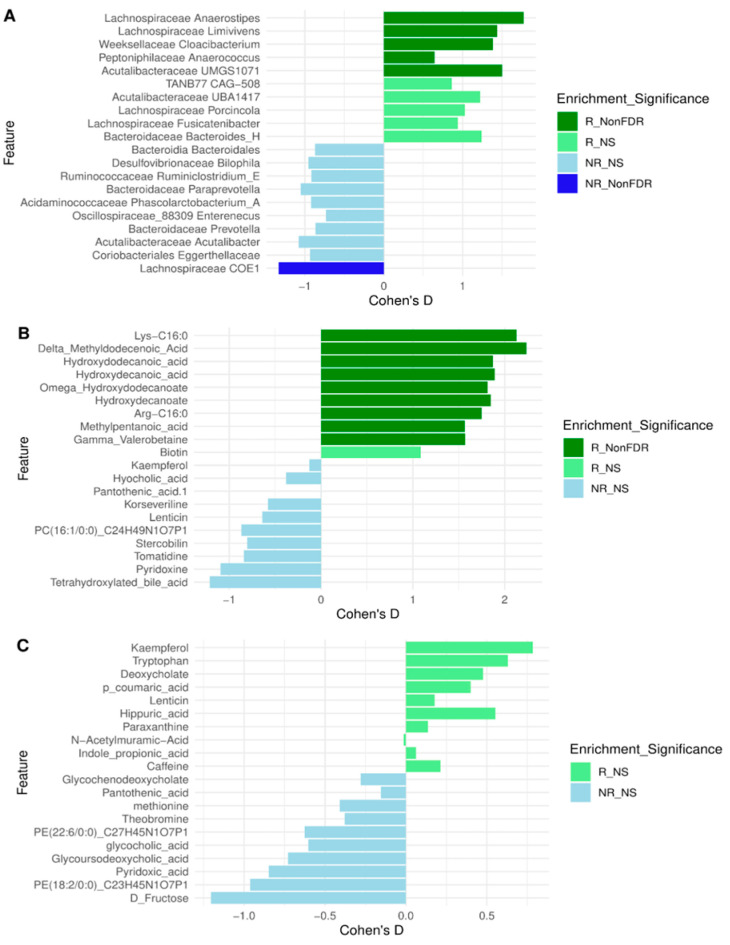
Comparison of changes in responders versus changes in non-responders for gut microbiome, gut metabolome, and plasma metabolome. Wilcoxon rank-sum tests comparing the distribution of post–pre abundances in responders versus non-responders for the gut microbiome (**A**), gut metabolome (**B**), and plasma metabolome (**C**). *R_FDR* indicates a positive change from pre- to post-intervention in responders compared to non-responders with q < 0.05 after FDR correction; *NR_FDR* indicates a positive change in non-responders with q < 0.05 after FDR correction. *R_NonFDR* indicates enrichment (*p* < 0.05) of a feature in responders not corrected by FDR. *NR_NonFDR* indicates enrichment (*p* < 0.05) in non-responders not corrected by FDR. NS indicates *p* > 0.05.

In the gut microbiome (Figure 7A), *Anaerostipes*, *Limivivens*, *Anaerococcus*, and *Cloacibacterium* (Weeksellaceae) showed greater increases in R compared to NR (*p* < 0.05), whereas *Lachnospiraceae* COE1 displayed a more pronounced decrease in R (*p* < 0.05).

In the gut metabolome (Figure 7B), several metabolites—including methyldodecanoic acid, lysine, hydroxydodecanoic acid, hydroxydecanoic acid, hydroxydecanoate, arginine, valerobetaine, and methylpentanoic acid—showed greater increases in R than in NR (*p* < 0.05).

By contrast, no significant differences were observed between R and NR in the plasma metabolome (Figure 7C), suggesting that systemic metabolic changes were not as strongly associated with dietary response.

### 3.8. Microbiome–Metabolome Network Changes over Time and Between Responders and Non-Responders

To investigate fecal microbiome–metabolome interactions between day 0 and 1 m, we applied DIABLO. Associations were visualized using differential chord diagrams, which maintain consistent node positioning to facilitate direct comparison of correlations across groups. This approach highlights differences in network structure between microbial taxa and metabolites.

**Figure 8:**
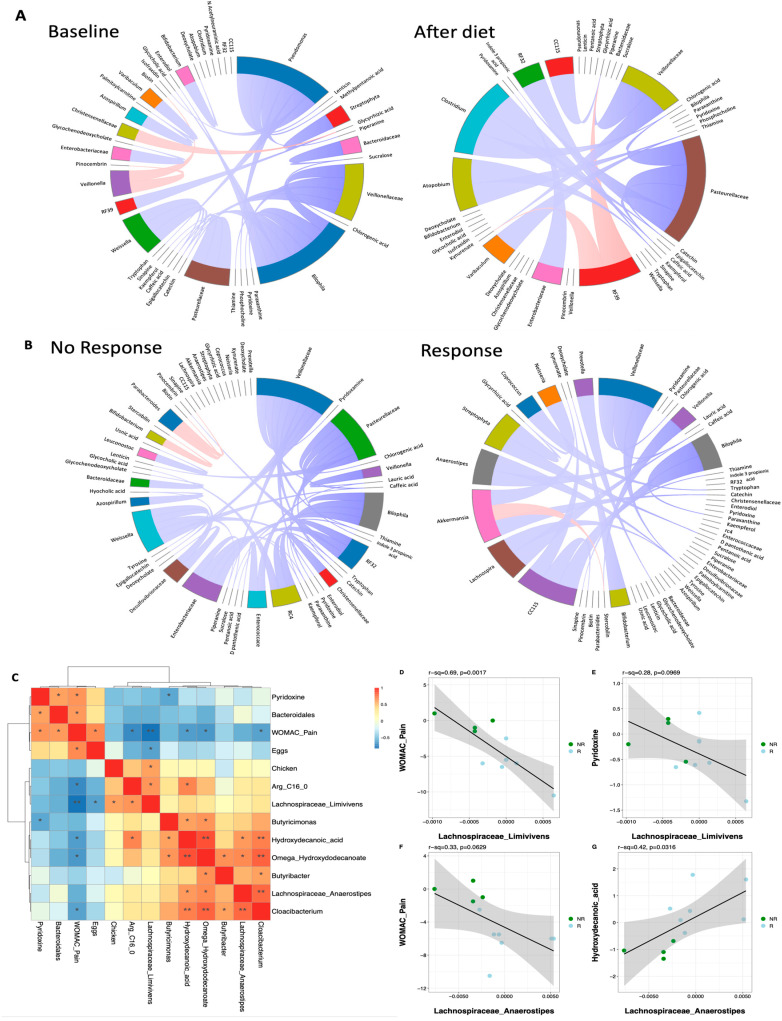
Chord diagrams reveal microbiome–metabolome differences over time and by response, with a heatmap of key associations. (**A**) Chord diagram showing microbiome–metabolome associations at baseline (left) and after diet (right); (**B**) chord diagram showing microbiome–metabolome associations at both timepoints for non-responders (left) and responders (right); (**C**) correlation matrix between differences in highlighted taxa, metabolites, diet elements, and clinical outcomes, where denotes * is *p* < 0.05 and ** is *p* < 0.01. (**D**–**G**) Scatterplots and Spearman correlation between (**D**) change in Lachnospiraceae Limivivens abundance and change in WOMAC pain, (**E**) change in Lachnospiraceae Limivivens abundance and change in pyridoxine levels, (**F**) change in Lachnospiraceae Anaerostipes abundance and change in WOMAC pain, and (**G**) change in Lachnospiraceae Anaerostipes abundance and change in hydroxydecanoic acid levels.

Figure 8A shows the integrated gut microbiome–metabolome networks at baseline (left) and post-intervention (right). At baseline, the network appeared more dispersed, with taxa such as *Veillonellaceae*, *Bilophila*, *Pseudomonas*, *Pasteurellaceae*, and *Weissella* displaying multiple interactions with metabolites. After four weeks, the network became more structured, with *Veillonellaceae*, *Clostridium*, and *Pasteurellaceae* still present but showing more focused interactions with specific metabolites.

Figure 8B compares microbiome–metabolome associations in NR (left) and R (right). In NR, the network was more diffuse, with taxa such as *Veillonellaceae*, *Bilophila*, *Enterobacteriaceae*, *Weissella*, and *Pasteurellaceae* engaging in multiple associations. In R, the network was denser, with taxa including *Akkermansia*, *Lachnospira*, *Anaerostipes*, *Streptophyta*, *Veillonellaceae*, *Bilophila*, *Bifidobacterium*, and *Prevotella* showing stronger connectivity. The circular layout separates microbes and metabolites for clarity but does not indicate data-driven clustering.

To summarize associations between microbial, metabolic, and clinical changes, we computed correlations between changes in taxa, metabolites, and WOMAC pain scores (Figure 8C). Notably, *Lachnospiraceae Limivivens* and *Lachnospiraceae Anaerostipes*—both enriched in responders—were associated with decreases in pain (*p* = 0.002 and *p* = 0.06, respectively) and with changes in pyridoxine (*p* = 0.10) and hydroxydecanoic acid (*p* = 0.03). Increased chicken intake and decreased egg intake were also linked to higher *Limivivens* abundance.

We observed a strong negative correlation between WOMAC pain improvement and changes in *Limivivens* (r = –0.83, *p* < 0.001; Figure 8D) and *Anaerostipes* (r = –0.56, *p* = 0.0016; Figure 8F). Except for *Anaerostipes*, these associations remained significant after adjusting for sex, race, BMI, and comorbidities (diabetes and hypertension) in generalized linear models (Supplementary Table S10).

### 3.9. Analysis of the Salivary Microbiome Revealed Limited Differential Features Before and After Dietary Intervention

In the salivary microbiome (Supplementary Figure S5A), we observed no significant post-intervention changes in alpha diversity (*p* = 0.266, Wilcoxon signed-rank) or beta diversity (*p* = 0.889, PERMANOVA). However, Veillonella and Nanosyncoccus were depleted after the dietary intervention.

In the salivary metabolome (Supplementary Figure S5B), neither alpha (*p* = 1) nor beta diversity (*p* = 0.798) changed, although nicotinuric acid and theobromine were depleted (*p* < 0.05).

When comparing baseline values between responders and non-responders, the salivary microbiome (Supplementary Figure S6A) showed no significant differences in alpha diversity (*p* = 0.955), beta diversity (*p* = 0.915), or individual genera. Similarly, the salivary metabolome (Supplementary Figure S6B) showed no significant alpha (*p* = 0.628) or beta diversity differences (*p* = 0.454), though coumaric acid was depleted in NR compared to R (*p* < 0.05).

Endpoint comparisons between R and NR were also largely nonsignificant. In the salivary microbiome (Supplementary Figure S7A), alpha (*p* = 1) and beta diversity (*p* = 0.779) did not differ between groups. The salivary metabolome (Supplementary Figure S7B) also showed no differences in alpha (*p* = 0.268) or beta diversity (*p* = 0.642).

Finally, when comparing trajectories from baseline to post-intervention (Supplementary Figure S8A), Weeksellaceae increased more in R than NR (*p* < 0.05), while Firmicutes Bacilli decreased more in R than NR (*p* < 0.05). No significant trajectory differences were observed in the salivary metabolome (Supplementary Figure S8B).

## 4. Discussion

A total of 21 OA patients completed the 4-week ITIS diet trial. Baseline diet logs revealed patients were consuming both pro-inflammatory foods, most notably red meat, forbidden/restricted beverages (alcohol, soda, coffee), and refined grains, as well as anti-inflammatory foods, including vegetables, berries, and enzymatic fruits. However, patients generally consumed few probiotics, PUFA, MUFA, or anti-inflammatory spices. The intervention was well-received, with an overall adherence rate of 66.20%. Participants increased their diet score from baseline, reflecting strong compliance with most prescribed dietary components. By the end of the 4-week intervention, patients showed notable clinical improvements in pain and function. A clinically meaningful 30% reduction in WOMAC pain identified eight participants as R and 12 as NR. Despite no baseline differences between groups before the diet intervention, R ultimately displayed lower post-diet WOMAC scores and other clinical scores, underscoring the potential of targeted dietary strategies to ameliorate OA-related symptoms.

Our findings align closely with outcomes reported in previous dietary intervention studies for OA, highlighting the effectiveness of targeted nutritional strategies in improving clinical symptoms. These results are consistent with previous research, such as the “Plants for Joints” study [48], which reported significant reductions in pain and stiffness alongside enhanced physical function after a 16-week multidisciplinary lifestyle program combining a plant-based diet, physical activity, and stress management. Similarly, the MD demonstrated significant reductions in pain and improvements in physical function in patients with knee OA over 12 weeks, independent of weight loss [49]. These findings collectively underscore the efficacy of dietary interventions in managing OA symptoms.

Adherence to the ITIS diet, as measured by dietary scores, increased from baseline (32.82 ± 28.65) to post-diet (140.35 ± 46.71, *p* < 0.001). One study reported a 96% adherence rate in a telehealth-delivered anti-inflammatory dietary intervention [50], while another reported an average adherence of 72% in a community-based, calorie-restricted diet and exercise program [51], more like our study. Of interest, in our study, both R and NR had similar adherence [R: 144.63 ± 21.18 vs. NR: 131.15 ± 24.68, *p* = 0.36], underscoring that other variables are more critical for the response to the ITIS diet.

A key insight from our study is that R had higher baseline consumption of anti-inflammatory dietary components, including whole grains, vegetables, cruciferous, berries and enzymatic fruit, other fruits, PUFA, MUFA, probiotics, and anti-inflammatory spices, emphasizing the importance of baseline food intake in achieving positive outcomes. Responders in the ITIS diet cohort exhibited higher baseline anti-inflammatory scores (81.99 ± 38.14) compared to NR (42.71 ± 14.00, *p* = 0.03), a pattern consistent with findings from our study in patients with RA [52].

Since baseline dietary patterns, rather than adherence, appeared to drive the response, we investigated whether the 4-week ITIS diet intervention was associated with notable shifts in the gut microbiome, metabolome, plasma metabolome, or salivary microbiome and metabolome. We first analyzed changes in the gut microbiome as a result of the intervention, aggregating all patients. We found that *Lachnospiraceae* (*Oliverpabstia*, *Fusicatenibacter*), *Ruminococcaceae* (*Phocea*) and *Clostridium A* had higher abundance at baseline than at post-intervention. After the dietary intervention, *Acetatifactor* increased in abundance. These findings are in line with the previous literature indicating a significant role for *Lachnospiraceae* and *Ruminococcaceae* families in the gut ecosystem. Specifically, the review by Vacca et al. [53] highlights that *Lachnospiraceae*, including genera such as *Fusicatenibacter*, play dual roles in health and disease. Their abundance is modulated by dietary interventions and can shift in response to an increased intake of plant-based nutrients. Moreover, the review supports that *Lachnospiraceae* genera are key players in metabolic interactions and inflammatory responses, and that some genera, such as *Roseburia* and *Blautia*, are positively associated with the production of beneficial metabolites like short chain fatty acids, which influence intestinal health and immune modulation. Patients also exhibited distinctly different plasma metabolomic profiles after the dietary intervention, with an enrichment of N-acetylmuramic acid, a component of bacterial cell walls, and indole-3-propionic acid, a known modulator of inflammation [54]. Additionally, we observed a depletion of theobromine, paraxanthine, vanillin, and caffeine, some of which associate with beverages (such as coffee) restricted in the ITIS diet.

When stratifying by responder status, we observed specific features in both the gut microbiome and plasma metabolome that were associated with the dietary intervention. The gut microbiome of R had higher post-intervention diversity, accompanied by increases in *Butyribacter* and *Akkermansia*, which are known to produce short-chain fatty acids [55]. These findings parallel those of a previous ITIS diet study in RA [52], suggesting a shared mechanism of clinical improvement across cohorts.

When comparing the changes in bacterial abundance from pre- to post-intervention between R and NR (as shown in Figure 7 and Figure 8), two genera from the *Lachnospiraceae* family—*Anaerostipes* and *Limivivens*—showed greater increases in abundance in the responder group. In other words, these bacteria shifted more in people who responded positively to the intervention. Additionally, both genera were negatively correlated with the change in WOMAC pain scores (Figure 8D–F), meaning that higher levels of these bacteria were associated with a greater reduction in pain, although neither of these taxa were differentially enriched between responders and non-responders at baseline or after the dietary intervention. This could be partially driven by the modest sample size, which limits the statistical power of our study. Nonetheless, our findings identified SCFA-producing and bile acid-metabolizing taxa, such as *Akkermansia*, *Butyribacter*, *Anaerostipes*, and *Limivivens*, as potential contributors to clinical response.

In the gut metabolome analysis, R showed a greater post-intervention increase in hydroxydecanoic acid and its derivatives compared to NR. This aligns with previous research showing that 10-hydroxydecanoic acid, a compound structurally related to the ones identified in our study, possesses potent anti-inflammatory properties. According to You et al. [56], 10-hydroxydecanoic acid suppresses LPS-induced inflammation in microglial cells by modulating the p53 pathway, inhibiting pro-inflammatory mediators (IL-6, TNF-α), and enhancing autophagy. Similarly, Chen et al. [57] demonstrated that 10-hydroxydecanoic acid reduced the production of inflammatory cytokines (nitric oxide, IL-6, IL-10) and modulated the MAPK and NF-κB pathways in LPS-stimulated macrophages, further supporting its potential role in inflammation resolution.

In addition to changes in the gut microbiome, gut metabolomics, and plasma metabolomics, our analysis of saliva revealed distinct baseline differences in both the microbiome and metabolome profiles. At baseline, the saliva microbiome showed a higher abundance of *Bacillus*, *Veillonella*, and *Nanosyncoccus*. While no existing research specifically examines the saliva microbiome in OA, a study in RA reported an increased relative abundance of *Prevotella* and *Veillonella* in the saliva of early RA patients and at-risk individuals, compared to healthy controls [58]. The metabolome analysis revealed higher levels of eudesmin, nicotinuric acid, and theobromine at baseline than after the diet intervention. We observed microbiome changes after the dietary intervention among R, with an increase in *Neisseriaceae* and *Weeksellaceae*. In addition, higher baseline concentrations of trihydroxylated bile acids, mangostin alpha, theobromine, and eudesmin were observed in the metabolome of R. A previous study on the saliva metabolome and its association with OA severity and synovitis in middle-aged women identified cystine, uric acid, and tyrosine as common metabolites linked to radiographic OA severity and effusion–synovitis [59]. However, these specific metabolites were not detected in our analysis, suggesting that variability might be partially due to the specific platforms used to quantify the metabolome.

Saliva-omics analyses in our cohort yielded null findings. Whole saliva may be a relatively insensitive matrix for detecting systemic or joint inflammation, particularly in cohorts with low periodontal disease burden and low inflammatory activity. Prior studies, especially in RA, have reported stronger and more consistent oral microbiome alterations when sampling subgingival plaque rather than saliva, with taxa such as *Porphyromonas gingivalis*, *Prevotella*, and *Veillonella* linked to systemic inflammatory markers, autoantibody production, and even bacterial product translocation to the joint space [60]. In OA, reported associations with the oral microbiome are more heterogeneous and generally weaker, though potential pathogenic taxa and functional pathways (e.g., lipopolysaccharide biosynthesis) have been identified in saliva [61]. The differences across oral niches, combined with the high short-term variability of salivary communities and the limitations of single-timepoint sampling, likely reduced our ability to detect consistent associations in this study.

This is the first study to investigate the effects of an anti-inflammatory diet while simultaneously analyzing the gut microbiome, gut microbial metabolome, plasma metabolome, salivary microbiome, and salivary metabolome in OA patients. This integrative approach provides a comprehensive understanding of the potential interactions between diet, microbial composition, and metabolic changes in OA. Although the low sample size and two-timepoint study design limit the power and ability to infer causality, these observations underscore the potential of dietary interventions to modulate the gut microbiome in OA patients, promoting SCFA-producing bacterial profiles that may contribute to improved clinical outcomes. Our findings, alongside prior evidence, highlight the intricate interplay between diet, gut microbiota, and the pathophysiology of OA.

### Limitations

This study has several limitations. First, it was an open-label pilot study with a small sample size (*n* = 21), which limits statistical power and generalizability. Second, the intervention period was relatively short (4 weeks), which may not capture long-term effects on pain, metabolic outcomes, or microbiome profiles. Changes in microbiota and metabolite composition observed within this timeframe may not have reached a new steady state, and it is unknown whether these effects would persist, diminish, or evolve with prolonged dietary intervention.

Third, the absence of a placebo or control group prevents fully separating the effects of the intervention from placebo responses, regression to the mean, or the natural course of symptoms. Placebo effects are well documented in pain research and can arise from participants’ expectations, increased clinical attention, or perceived novelty of the intervention, leading to symptom improvement independent of the intervention’s biological mechanism. Regression to the mean is also a concern in studies enrolling participants with high baseline pain, as extreme values tend to move closer to the population average upon repeated measurement, regardless of intervention. This is particularly relevant for subjective pain measures such as the WOMAC pain score, which are inherently susceptible to expectation bias. Although we applied clear inclusion and exclusion criteria and adjusted for demographic and clinical covariates (e.g., sex, BMI, diabetes) in generalized linear models, the lack of blinding and a control arm remains an important consideration when interpreting results.

Fourth, the study population consisted of a single, non-randomized cohort without replication across independent subjects or study centers, which may limit representativeness for the broader OA population. These findings should therefore be considered exploratory and hypothesis-generating. While we employed appropriate statistical methods for small sample sizes and non-normal distributions, we did not comprehensively test all assumptions (e.g., independence, normality, or homogeneity of variance), which may affect the validity of some inferences.

Finally, although dietary intake was monitored and participants received guidance to follow the assigned dietary protocol, variability in adherence and unmeasured differences in baseline dietary patterns cannot be ruled out. Without direct measures of adherence or objective biomarkers of dietary intake, it is difficult to isolate the specific effects of the intervention from broader behavioral or contextual influences, such as increased attention or motivation from study participation. These factors, which are common in nutrition intervention studies, should be addressed in future randomized trials with objective adherence assessments and broader contextual controls.

## Data Availability

The microbiome sequencing data generated and analyzed in this study have been deposited in the Short Read Archive of NCBI BioProject: PRJNA1159155. The metabolomics data reported was uploaded on GNPS: blood plasma (plasma), saliva (saliva) and stool (stool). All MS data (.d and .mzXML files) are publicly available via GNPS/MassIVE (massive.ucsd.edu), a public MS data repository, under the accession number MSV000094097.

## Supplementary Materials

The following supporting information can be downloaded at: https://www.mdpi.com/article/10.3390/nu17172729/s1, Figure S1: STROBE flow diagram of patient inclusion and exclusion criteria; Figure S2: Genus level taxa bar plots; Figure S3: Additional dietary-related changes by response at baseline and after diet; Figure S4: Association of endpoint gut microbiome, gut metabolome, and plasma metabolome features with responder status; Figure S5: Impact of diet on salivary microbiome and metabolome irrespective of responder status; Figure S6: Association of baseline saliva microbiome and saliva metabolome features with responder status; Figure S7: Association of endpoint saliva microbiome and saliva metabolome features with responder status; Figure S8: Comparison of changes in responders versus changes in non-responders for salivary microbiome and metabolome; Table S1: Feasibility outcomes of the trial; Table S2: Summary of dietary recommendations; Table S3: Proposed meal organization for the 2 weeks of the intervention; Table S4: Diet score calculation; Table S5: Clinical outcomes on day-14 and day 0; Table S6: Change in diet scores after diet; Table S7: Outcomes differences between responders and non-responders at baseline; Table S8: Changes in diet scores after diet by response to Womac pain; Table S9: Change in diet scores differences between responders and non-responders at baseline and after diet; Table S10: Generalized linear models. Refs. [38,47,62,63,64,65,66,67] are from Supplementary Materials.

## Abbreviations

The following abbreviations are used in this manuscript:

BER	Balanced error rate
BMI	Body mass index
CES-D	Center for Epidemiological Studies Depression
DIABLO	Data Integration Analysis for Biomarker Discovery Using Latent Components
FA	Fatty acids
FDR-BH	False Discovery Rate Using the Benjamini–Hochberg
IL	Interleukin
LC-MS	Liquid chromatography–mass spectrometry
LPS	Lipopolysaccharide
MAPK	Mitogen-activated protein kinase
MD	Mediterranean diet
MS	Mass spectrometry
MUFA	Monounsaturated fatty acids
NF-κB	Nuclear factor kappa B
NR	Non-responders
OA	Osteoarthritis
PASE	Physical Activity Scale for the Elderly
PCoA	Principal coordinate analysis
PCS	Pain catastrophizing scale
PERMANOVA	Permutational multivariate analysis of variance
PUFA	Polyunsaturated fatty acids
R	Responders
RA	Rheumatoid arthritis
rCLR	Robust-centered log ratio
SCFA	Short-chain fatty acids
SD	Standard deviation
TNF	Tumor necrosis factor
VAS	Visual analog scale
WOMAC	Western Ontario and McMaster Universities Arthritis Index
