# Supplementary material for: Targeted Microbial Shifts and Metabolite Profiles Were Associated with Clinical Response to an Anti-Inflammatory Diet in Osteoarthritis
Source: Nutrients. 2025 Aug 22;17(17):2729. doi: 10.3390/nu17172729 (PMC12430150; doi:10.3390/nu17172729)
Supplement: Supplementary file 1 [file nutrients-17-02729-s001.zip › Supplementary Figures/SFigure_legends.pdf]

SUPPLEMENTARY FIGURE LEGENDS

Supplementary Figure S1: STROBE flow diagram of patient inclusion and exclusion criteria. The diagram summarizes the stepwise process of patient selection for the study, including the number of individuals assessed for eligibility, excluded due to predefined criteria, and the final number included in the analysis.

Supplementary Figure S2: Genus level taxa barplots for stool samples (top) and salivary samples (bottom), separated by pre- and post- dietary intervention (left and right, respectively). Top fifteen most abundant genera are shown with the remainder in light gray.

Supplementary Figure S3. Additional dietary-related changes by response at baseline and after diet. A) Whole grains score changes after the dietary intervention by response; **B)** Cruciferous score consumption score before and after diet by response; **C)** Vegetable intake score at baseline and after diet by response; **D)** Other fruits score variation before and after diet by response; **E)** Probiotics score evolution over time by response; **F)** Anti-inflammatory spices score before and after diet by response.

Supplementary Figure S4. Association of endpoint gut microbiome, gut metabolome, and plasma metabolome features with responder status. Alpha diversity, beta diversity, and differential abundance analyses for the gut microbiome (A), gut metabolome (B), and plasma metabolome (C) at endpoint comparing responders and non-responders. In the leftmost column, Wilcoxon Rank-sum tests were conducted for differences in alpha diversity, PERMANOVA was used for assessing differences in composition in the center column, and Wilcoxon Rank-sum tests were used to identify differential features in the rightmost column. R_NonFDR indicates an enrichment of said feature in responders with $p<0.05$, while R_NS indicates $p>0.05$. NR_NonFDR and NR_NS indicates the analogous significance levels for enrichment in the nonresponder cohort.

Supplementary Figure S5. Impact of diet on salivary microbiome and metabolome irrespective of responder status. Alpha diversity, beta diversity, and differential abundance analyses for saliva microbiome (A) and saliva metabolome (B). In the leftmost column, paired Wilcoxon Signed-Rank tests were conducted for differences in alpha diversity, PERMANOVA was used for assessing differences in composition in the center column, and paired Wilcoxon Signed-Rank tests were used to identify differential features in the rightmost column. Post_FDR indicates an enrichment of said feature in post-intervention samples with $q<0.05$, while Post_NonFDR indicates $p<0.05$ and Post_NS indicates $p>0.05$.

Pre_FDR, Pre_NonFDR, and Pre_NS indicates the analogous significance levels for enrichment in the pre-intervention samples.

Supplementary Figure S6. Association of baseline saliva microbiome and saliva metabolome features with responder status. Alpha diversity, beta diversity, and differential abundance analyses for the saliva microbiome (A) and saliva metabolome (B) comparing baseline values between responders and non-responders. In the leftmost column, Wilcoxon Rank-sum tests were conducted for differences in alpha diversity, PERMANOVA was used for assessing differences in composition in the center column, and Wilcoxon Rank-sum tests were used to identify differential features in the rightmost column. R_NonFDR indicates an enrichment of said feature in responders with $p<0.05$, while R_NS indicates $p>0.05$. NR_NonFDR and NR_NS indicates the analogous significance levels for enrichment in the nonresponder cohort.

Supplementary Figure S7. Association of endpoint saliva microbiome and saliva metabolome features with responder status. Alpha diversity, beta diversity, and differential abundance analyses for the saliva microbiome (A) and saliva metabolome (B) comparing endpoint values between responders and non-responders. In the leftmost column, Wilcoxon Rank-sum tests were conducted for differences in alpha diversity, PERMANOVA was used for assessing differences in composition in the center column, and Wilcoxon Rank-sum tests were used to identify differential features in the rightmost column. R_NonFDR indicates an enrichment of said feature in responders with $p<0.05$, while R_NS indicates $p>0.05$. NR_NonFDR and NR_NS indicates the analogous significance levels for enrichment in the nonresponder cohort.

Supplementary Figure S8. Comparison of changes in responders versus changes in non-responders for salivary microbiome and metabolome. Wilcoxon Rank-sum tests comparing the distribution of post-pre abundances in responders versus non-responders for the salivary microbiome (A) and metabolome (B). R_NonFDR indicates a more positive change from pre to post of said feature in responders compared to non-responders with $p<0.05$, while R_NS indicates $p>0.05$. NR_NonFDR and NR_NS indicates the analogous significance levels for a more positive difference (pre to post) in the nonresponder cohort.
