# Supplementary material for: Targeted Microbial Shifts and Metabolite Profiles Were Associated with Clinical Response to an Anti-Inflammatory Diet in Osteoarthritis
Source: Nutrients. 2025 Aug 22;17(17):2729. doi: 10.3390/nu17172729 (PMC12430150; doi:10.3390/nu17172729)
Supplement: Supplementary file 1 [file nutrients-17-02729-s001.zip › Supplementary Tables/Supplementary Tables_6.6.2025.pdf]

Supplementary Table S1: Feasibility outcomes of the trial

**Screening**	
Patients screened	60
Number excluded	26
Reasons for exclusion	Unable to contact -6<br>Decided not to start the trial once they knew more details about it or drop out - 5<br>Other diagnosis - 2<br>Not eligible - 4<br>Too busy - 7<br>Only if financial compensation - 2
Motivation of the patient to be part of the study	Did not want to take more pills<br>Did not want to escalate therapy as they were afraid of side-effect<br>Believed that diet could help and wanted to try this first
**Enrollment**	
Number consented and enrolled	34
Number completed baseline assessments / bio-specimens	34
Number withdrawn	13
Reasons for withdrawal	6 – lost– unable to contact<br>7 – decided to stop after seeing the diet on d0 – too difficult to follow
Number completed protocol	21
Number currently active	0
**Adverse events**	
Number of adverse events	0

Supplementary Table S2. Summary of dietary recommendations

Main recommendations (WHAT)	Diet strategies (HOW)
Increase omega 3 lipid intake and decrease omega 6 lipid intake (red meat, frying and refined oils, pre-cooked food)	The diet must contain oily fish such as two days in the week
	Daily intake of avocado and or sesame seeds or tahini
	Condiment with flaxseed oil
Increase the consumption of green leafy vegetables and fruits	Daily green leafy vegetables (arugula, lettuce, broccoli, zucchini, Green beans)
	Daily home-made Green juice (made of fruits and green vegetables
Introduce the consume of prebiotics and probiotics	Daily yogurt or miso
To reinforce particular types of cooking	Recommendation of steam cooking, baking and not too long boiling, and decrease frying and long boiling processes
To introduce enzymatic fruits	Daily enzymatic fruit (pineapple, mango or papaya) as a snack
To eliminate sauces and introduce anti-inflammatory condiments	Condiment with turmeric and/or black pepper
To decrease solanaceae vegetables and introduce vegetables with anti-inflammatory properties	Forbidding the consumption of eggplant, tomatoes and potatoes and suggest consumption of garlic, onion, carrot, pumpkin, zucchini or others
To substitute red meat per other types of proteins	Reduce animal meat to chicken or turkey to twice a week
	Two-three times a week, introduce legumes (red, white beans, lentils or garbanzo)
	Two-three eggs per week
	Two-three days per week, eat fish
To reduce the consumption of gluten	To reduce the intake of wheat bread and change it for rye bread or corn tortillas
To delete dairy products (except yogurt) due its content of large proteins	To forbid dairy products and to suggest consumption of vegetable milks (almond, rice, coconut)
To introduce chia seeds, since they contain high quantities of tryptophan, serotonin precursor associated with control of pain	Suggested for salads and/or juices
To maintain a good acidic-basic balance by introducing alkalinizing juices	Daily home-made Green juice (made of fruits and green vegetables)

Supplementary Table S3. Proposed meal organization for the 2 weeks of the intervention

		DAY 1 and 8	DAY 2 and 9	DAY 3 and 10	DAY 4 and 11	DAY 5 and 12	DAY 6 and 13	DAY 7 and 14
**7-8 am**	**SMOOTHIE**	Coconut milk, mango, papaya, pineapple	Pear, lemon, yogurt, vanilla, water	Grapes, celery, spinach, cucumber, lime, water	Almond or oat milk, spinach, strawberries, pear, chia seeds, cinnamon and ginger	Spinach, ginger, turmeric, papaya, flaxseeds, banana, water	Papaya, spinach, almond milk, turmeric, chia seeds, honey	Parsley, pineapple, strawberries, water
**7-8 am**	**BREAKFAST**	1-2 spoons of oats with non-dairy milk (oat milk, almond milk or rice milk). Add berries (optional).	1-2 corn tortillas, spread with avocado, sesame seeds, and flaxseed oil.	1 -2 corn tortillas with tahini (sesame seed extract) with ¼ teaspoon of honey .	1-2 spoons of oat with non-dairy milk (oat milk, almond milk or rice milk. Add berries (optional).	1-2 corn tortillas, spread with avocado, sesame seeds, and flaxseed oil.	1 -2 corn tortillas with tahini (sesame seed extract) with ¼ teaspoon of honey.	1-2 corn tortillas, spread with avocado, sesame seeds, and linseed oil.
		Green tea infusion.	Green tea infusion.	Green tea infusion	Green tea infusion.	Green tea infusion.	Green tea infusion	Green tea infusion.
**10-11 am**	**SNACK**	Plain yogurt (Chobani Brand, no sugar added)	Plain yogurt (Chobani Brand, no sugar added)	Plain yogurt (Chobani Brand, no sugar added)	Plain yogurt (Chobani Brand, no sugar added)	Plain yogurt (Chobani Brand, no sugar added)	Plain yogurt (Chobani Brand, no sugar added)	Plain yogurt (Chobani Brand, no sugar added)
**12-1 pm**	**LUNCH**							
		OPTION 1: Salad (generous plate)						
		OPTION 2: Grains with vegetables						
		OPTION 3: Legumes with vegetables						
**4:00 PM**	**SNACK**	Mango, papaya, pineapple, apple, pear or banana + 4 walnuts	Mango, papaya, pineapple, apple, pear or banana + 4 walnuts	Mango, papaya, pineapple, apple, pear or banana + 4 walnuts	Mango, papaya, pineapple, apple, pear or banana + 4 walnuts	Mango, papaya, pineapple, apple, pear or banana + 4 walnuts	Mango, papaya, pineapple, apple, pear or banana + 4 walnuts	Mango, papaya, pineapple, apple, pear or banana + 4 walnuts
**7-8 pm**	**DINNER**							
		OPTION 1: Vegetable soup/cream + protein						
		OPTION 2: Miso soup + baked/steamed/grilled vegetables + protein						
		OPTION 3: Salad + protein						

Supplementary Table S4. Diet score calculation

Food groups	Food category	Score	What to do if they eat more
**Protein**	Poultry (3 times a week)	6	Max 6
	Red meat	0	Subtract if > 0
	White or Fatty fish (3 times a week, per 2)	12	Max 12
	Seafood	0	Subtract if > 2
	Legumes (3 times a week)	6	Max 6
	Eggs	0	if > 4 /week - subtract
	Total	**24**	
**Bread and cereals**	Whole grains	8	Max 8
	Refined grains	0	Subtract if > 0
	Pseudocereals	4	Max 4
	Oats	4	Max 4
	Biscuits, white bread, other cereals	0	Subtract if > 0
	Total	**16**	
**Vegetables&mushrooms**	Greens (includes lettuce, spinach, other types of green leafy vegetables,)	8	Max 8
	Nongreens (celery, peppers, cucumber, artichoke, onion, radishes, carrot…)	8	Max 8
	Cruciferous (cauliflower, cabbage, kale, garden cress, bok choy, broccoli, Brussels sprouts)	16	Max 16
	Tomato	0	Subtract if > 0
	Potato	0	Subtract if > 0
	Eggplant	0	Subtract if > 0
	Total	**32**	
**Fruit**	Berries	8	Max 8
	Other fruits	14	Max 14
	Enzymatic fruit	14	Max 14
	Total	**36**	
**Fat**	Saturated (precooked, processed food, butter)	0	Subtract if > 0
	Polyunsaturated (flaxseeds oil, seeds) (daily)	14	Max 14

	Monounsaturated (tahini or avocado) (daily)	14	Max 14
	Total	**28**	
**Nuts**	Walnuts	14	Max 14
	Total	**14**	
**Miso**	Miso (4 times x 3)	12	Max 12
**Dairy**	Milk and derivates	0	Subtract if > 0
	Plain Yogurt	14	Max 14
	Flavored yogurt	0	Subtract if > 0
	Total	**14**	
**Drinks**	Coffee	0	Subtract if > 0
	Alcohol	0	Subtract if > 0
	Green tea (daily)	14	Max 14
	Sweetened beverages (Soda, energy drinks, fruit drinks)	0	Subtract if > 0
	Total	**14**	
**Added sugars**	Sugar, pastries	**0**	Subtract if > 0
**Sauces/spices**	Turmeric - Ginger- Black Pepper (14 per turmeric/black pepper/daily, 4 per ginger)	18	Max 18
	Cinnamon	2	
	Vanilla	2	
	Sauces	0	Subtract if > 0
	Total	**22**	
**Total**		212	

Supplementary Table S5. Clinical outcomes on day-14 and day 0

Variable	Day-14	Day0	P value
**VAS_PT (n=21)**	4.33±2.50	4.53± 2.63	0.77
**VAS_Overall (n=21)**	4.20±2.27	4.12±2.67	0.91
**WOMAC pain (n=20)**	11.43±3.37	10.95 ± 3.05	0.67
**WOMAC stiffness (n=20)**	4.28±1.73	4.50 ± 1.73	0.68
**WOMAC activity (n=20)**	37.22±11.40	36.85±12.09	0.92
**WOMAC total (n=20)**	52.93±14.10	52.30 ± 15.71	0.90
**PainDETECT(n=21)**	12.52±7.03	13.67 ± 6.82	0.59
**CES_D (n=21)**	16.76±9.40	19.57±7.52	0.49
**Helplessness (n=21)**	5.33±5.72	5.00±5.39	0.84
**Magnification (n=21)**	2.85±3.07	2.61±2.53	0.79
**Rumination (n=21)**	4.61±4.95	5.09±5.04	0.76
**PCS_EN (n=21)**	12.81±13.14	12.86±12.30	0.99
**Sleep_quality (n=21)**	24.47±7.91	21.95 ± 7.66	0.32
**BMI (n=14)**	30.97±8.87	31.55±10.04	0.97

Supplementary Table S6. Change in diet scores after diet

Food	Day 0	4 weeks	p value	Gold Standard
**Pro-inflammatory foods**				
**Animal protein**	-6.71 ± 6.3	-1.37 ± 1.96	**0.001**	0
**Refined grains**	-10.29 ± 11.43	-2.78 ± 3.76	**0.01**	0
**Solanaceae**	-2.97 ± 3.2	-0.5 ± 1.82	**0.01**	0
**Saturated fat**	0 ± 0	-0.1 ± 0.45	1	0
**Milk derivates**	-7.92 ± 10.03	-0.6 ± 1.85	**0.002**	0
**Beverages prohibited**	-16.69 ± 16.32	-1.97 ± 3.19	**0.002**	0
**Pro-inflammatory spices**	-3.3 ± 4.47	-0.8 ± 1.51	**0.03**	0
**Total pro inflammatory score**	-29.71 ± 26.58	-5.85 ± 6.44	**< 0.001**	0
**Anti-inflammatory**				
**Chicken**	3.19 ± 2.81	3.8 ± 2.67	0.36	6
**Plant protein**	1.5 ± 2.44	3.84 ± 2.48	**0.001**	6
**Whole grains**	7.28 ± 5.24	9.52 ± 3.96	0.12	16
**Vegetables**	7.67 ± 5.46	11.58 ± 4.81	**0.009**	16
**Cruciferous**	2.9 ± 4.22	12.87 ± 5.84	**< 0.001**	16
**Berries and enzymatic fruit**	7.82 ± 7.68	18.14 ± 6.65	**0.001**	22
**Other fruit**	2.43 ± 3.69	4.75 ± 4.22	**0.02**	14
**Blue fish**	3.44 ± 5.08	5.63 ± 4.9	0.14	12
**PUFA**	5.63 ± 5.16	12 ± 4.35	**0.001**	14
**MUFA**	2.02 ± 4.09	7.63 ± 4.66	**0.003**	14
**Walnuts**	0.42 ± 1.37	7.28 ± 5.09	**<0.001**	14
**Probiotics**	1.73 ± 3.59	15.86 ± 10.39	**< 0.001**	26
**Green tea**	1.68 ± 4.3	3.55 ± 4.68	0.22	14
**Anti-inflammatory spices**	2.22 ± 5.26	14.33 ± 7.72	**< 0.001**	22
**Total anti-inflammatory score**	62.53 ± 34.08	146.20 ± 21.80	**< 0.001**	212
**Total score**	32.82 ± 28.65	140.35 ± 46.71	**< 0.001**	212

Supplementary Table S7. Outcomes differences between responders and no responders at baseline

	Baseline		
	**Response (n=8)**	**No response (n=12)**	
**Visit**	d-15/d0		P-value
**BMI**	28.39 ± 5.96	32.72 ± 10.65	0.51
**VAS_Pt**	5.06 ± 1.92	4.19 ± 2.15	0.24
**VAS_Overall**	4.12 ± 2.35	4.37 ± 2.16	0.90
**WOMAC_Pain**	12.13 ± 2.63	10.56 ± 2.82	0.2
**WOMAC_stiffness**	4.78 ± 1.37	4.13 ± 1.63	0.48
**WOMAC_activity**	39.72 ± 9.33	35.25 ± 11.95	0.37
**WOMAC_total**	56.63 ± 12.65	49.94 ± 14.7	0.26
**Pain_Detect**	13.5 ± 6.21	12.25 ± 7.13	0.93
**CES_D**	20.19 ± 7.92	17.46 ± 8.06	0.27
**Helplesness**	4.69 ± 4.78	5.79 ± 5.5	0.56
**Magnification**	2.81 ± 2.98	2.88 ± 2.56	0.90
**Rumination**	4.94 ± 4.29	5.17 ± 5.27	0.96
**PCS_EN**	12.56 ± 11.64	13.96 ± 12.96	0.87
**Sleep_disturbance**	26.5 ± 7.28	21.21 ± 7.88	0.17
**PASE_walk**	27.35 ± 18.01	14.66 ± 14.34	0.09
**PASE_light**	13.12 ± 20.35	11.06 ± 18.28	0.69
**PASE_moderate**	0.92 ± 2.6	6.98 ± 13.53	0.43
**PASE_strenuous**	7.71 ± 20.79	15.39 ± 23.35	0.44
**PASE_muscle**	12.19 ± 26.67	12.56 ± 15.09	0.43

Supplementary Table S8. Changes in diet scores after diet by response to Womac Pain.

	Response (n=8)			No response (n=12)		
	**Baseline**	**After diet**	**P value**	**Baseline**	**After diet**	**P value**
**red_animal**	-7.38 ± 5.31	-1.63 ± 2.72	**0.02**	-5.81 ± 7.25	-1.12 ± 1.4	0.09
**refined_grains**	-10.59 ± 11.29	-2.54 ± 2.56	**0.01**	-8.96 ± 11.93	-3.2 ± 4.61	0.18
**slanaceae**	-4.48 ± 3.32	-0.25 ± 0.71	**0.03**	-1.89 ± 2.94	0 ± 0	0.1
**saturated**	0 ± 0	-0.25 ± 0.71	1	0 ± 0	0 ± 0	1
**milk_derivates**	-9.06 ± 7.37	-0.25 ± 0.71	**0.02**	-6.79 ± 12.23	-0.18 ± 0.6	**0.05**
**prohibited_beverages**	-17.8 ± 13.94	-1.42 ± 1.79	**0.008**	-16.38 ± 19.14	-1.82 ± 3.63	**0.05**
**inflammatory_spices**	-5.11 ± 5.03	-0.5 ± 0.93	**0.03**	-2.29 ± 3.92	-0.73 ± 1.62	0.19
**inflammatory_total**	-59.98 ± 24.32	-7.08 ± 6.64	**0.008**	-47.46 ± 51.22	-7.75 ± 8.23	**0.03**
**chicken**	4.03 ± 2.68	4.75 ± 2.12	0.52	2.33 ± 2.81	2.91 ± 2.88	0.67
**plant_protein**	0.35 ± 0.99	4.5 ± 2.33	**0.02**	2.48 ± 2.87	3.35 ± 2.7	**0.03**
**whole_grains**	10.66 ± 4.23	10.67 ± 1.17	1	5.13 ± 4.93	8.45 ± 5.09	0.15
**vegetables**	10.22 ± 3.86	14.08 ± 2.32	**0.03**	5.78 ± 6.1	10.08 ± 5.6	0.07
**cruciferous**	5.15 ± 4.42	12.17 ± 5.54	**0.05**	1.02 ± 3.38	13.09 ± 6.47	**0.005**
**berries_enzymatic_fruit**	11.43 ± 7.68	19.75 ± 2.92	**0.05**	3.9 ± 4.83	16.62 ± 8.48	**0.01**
**other_fruit**	5.26 ± 4.45	6.04 ± 5.09	0.61	0.6 ± 1.13	3.7 ± 3.6	**0.01**
**fatty_fish**	5.1 ± 5.82	5.67 ± 4.87	0.85	2.55 ± 4.59	5.03 ± 4.95	0.35
**PUFA**	10.45 ± 3.3	13.5 ± 1.41	0.09	2.63 ± 3.36	10.73 ± 5.53	**0.01**
**MUFA**	5.05 ± 5.29	9.54 ± 4.55	0.14	0 ± 0	6.39 ± 4.67	**0.009**
**Walnuts**	1.05 ± 2.08	8.71 ± 4.62	**0.02**	0 ± 0	6.18 ± 5.6	**0.02**
**probiotics**	4.2 ± 4.96	19.5 ± 6.82	**0.02**	0.23 ± 0.81	12.59 ± 11.67	**0.009**
**green_tea**	3.85 ± 6.34	4.88 ± 5.22	0.83	0.25 ± 0.84	1.82 ± 3.28	0.27
**antiinflammatory_spices**	5.2 ± 7.57	17.96 ± 4.84	**0.02**	0.25 ± 0.84	12.26 ± 8.81	**0.009**
**antiinflammatory_score**	81.99 ± 38.14	151.71 ± 19.89	**0.008**	42.71 ± 14	139.77 ± 23.72	**0.03**
**Total_score**	22.01 ± 58.62	144.63 ± 21.18	**0.01**	-19.51 ± 46.68	131.15 ± 24.68	**0.008**

Supplementary Table S9. Change in diet scores differences between responders and no responders at baseline and after diet

	Baseline			4 Weeks			
**Food**	**Responder (n=8)**	**No responder (n=12)**	**p value**	**Responder (n=8)**	**No responder (n=12)**	**p value**	**Gold standard**
**Pro-inflammatory foods**							
**Animal protein**	-7.38 ± 5.31	-5.81 ± 7.25	0.5	-1.63 ± 2.72	-1.12 ± 1.40	1	0
**Refined grains**	-10.59 ± 11.29	-8.96 ± 11.93	0.42	-2.54 ± 2.56	-3.2 ± 4.61	0.69	0
**Solanaceae**	-4.48 ± 3.32	-1.89 ± 2.94	0.08	-0.25 ± 0.71	0 ± 0	0.28	0
**Saturated fat**	0 ± 0	0 ± 0	1	-0.25 ± 0.71	0 ± 0	1	0
**Milk derivates**	-9.06 ± 7.37	-6.79 ± 12.23	0.1	-0.25 ± 0.71	-0.18 ± 0.60	0.877	0
**Beverages prohibited**	-17.80 ± 13.94	-16.38 ± 19.14	0.56	-1.42 ± 1.79	-1.82 ± 3.63	0.67	0
**pro inflammatory score**	-59.98 ± 24.32	-47.46 ± 51.22	0.37	-7.08 ± 6.64	-7.75 ± 8.23	0.92	0
**Anti-inflammatory**							
**Chicken**	4.03 ± 2.68	2.33 ± 2.81	0.27	4.75 ± 2.12	2.91 ± 2.88	0.18	6
**Plant protein**	0.35 ± 0.99	2.48 ± 2.87	0.09	4.50 ± 2.33	3.35 ± 2.70	0.39	6
**Whole grains**	10.66 ± 4.23	5.13 ± 4.93	**0.03**	10.67 ± 1.17	8.45 ± 5.09	0.42	16
**Vegetables**	10.22 ± 3.86	5.78 ± 6.10	0.06	14.08 ± 2.32	10.08 ± 5.60	0.07	16
**Cruciferous**	5.15 ± 4.42	1.02 ± 3.38	**0.01**	12.17 ± 5.54	13.09 ± 6.47	0.59	16
**Berries and enzymatic fruit**	11.43 ± 7.68	3.90 ± 4.83	**0.03**	19.75 ± 2.92	16.62 ± 8.48	0.63	22
**Other fruit**	5.26 ± 4.45	0.60 ± 1.13	**0.004**	6.04 ± 5.09	3.70 ± 3.60	0.35	14
**Fish**	5.10 ± 5.82	2.55 ± 4.59	0.25	5.67 ± 4.87	5.03 ± 4.95	0.89	12
**PUFA**	10.45 ± 3.30	2.63 ± 3.36	**0.001**	13.50 ± 1.41	10.73 ± 5.53	0.24	14
**MUFA**	5.05 ± 5.29	0.00 ± 0.00	**0.004**	9.54 ± 4.55	6.39 ± 4.67	0.15	14
**Walnuts**	1.05 ± 2.08	0.00 ± 0.00	0.1	8.71 ± 4.62	6.18 ± 5.60	0.45	14
**Probiotics**	4.20 ± 4.96	0.23 ± 0.81	**0.01**	19.50 ± 6.82	12.59 ± 11.67	0.22	26
**Green tea**	3.85 ± 6.34	0.25 ± 0.84	0.13	4.88 ± 5.22	1.82 ± 3.28	0.19	14
**Anti-inflammatory spices**	5.02 ± 7.57	0.25 ± 0.84	**0.03**	17.96 ± 4.84	12.26 ± 8.81	0.14	22
**Anti-inflammatory score**	81.99 ± 38.14	42.71 ± 14.00	**0.03**	151.71 ± 19.89	139.77 ± 23.72	0.33	212
**Total score**	22.01 ± 58.62	-19.51 ± 46.68	0.1	144.63 ± 21.18	131.15 ± 24.68	0.36	212

Supplementary Table S10. Generalized Linear Models

Linear models relating the *change* in WOMAC_Pain to the *change* (post minus pre) in specified features. Gaussian noise assumption. No patients had a comorbidity of diabetes.

glm(WOMAC_Pain ~ Plasma_Metabolome_Alpha_Diversity + Gut_Microbiome_Alpha_Diversity + HTN + Race + Sex)

**Coefficient**	**Estimate**	**Std. Err**	**T**	**P**
(Intercept)	21.5	4.1	5.2	0.014
Plasma Metabolome Alpha Diversity	15.8	2.0	7.9	0.004**
Gut Microbiome Alpha Diversity	9.6	2.3	4.3	0.024*
HTN	-0.9	0.7	-1.2	0.319
Hispanic	-23.1	3.8	-6.1	0.009
White	-25.2	4.0	-6.1	0.008
Male	-1.9	0.7	-2.8	0.07

glm(WOMAC_Pain ~ Gut_Lachnospiraceae_Limivivens + HTN + Race + Sex)

**Coefficient**	**Estimate**	**Std. Err**	**T**	**P**
(Intercept)	-1.3	2.6	-0.5	0.65
Gut Lachnospiraceae Limivivens	-6401	1824	-3.5	0.02*
HTN	-0.7	1.6	-0.4	0.70
Hispanic	-5.9	2.8	-2.1	0.10
White	-3.6	2.2	-1.6	0.18
Male	0.8	1.5	-0.5	0.62

glm(WOMAC_Pain ~ Gut_Lachnospiraceae_Anaerostipes + HTN + Race + Sex)

**Coefficient**	**Estimate**	**Std. Err**	**T**	**P**
(Intercept)	-4.1	6.5	-0.6	0.57
Gut Lachnospiraceae Limivivens	-158	486	-3.3	0.76
HTN	2.3	2.9	0.8	0.47
Hispanic	-3.9	7.5	-0.5	0.63
White	-0.9	5.8	-0.2	0.88
Male	0.5	3.3	0.2	0.88
