# Supplementary material for: Targeted Microbial Shifts and Metabolite Profiles Were Associated with Clinical Response to an Anti-Inflammatory Diet in Osteoarthritis
Source: Nutrients. 2025 Aug 22;17(17):2729. doi: 10.3390/nu17172729 (PMC12430150; doi:10.3390/nu17172729)
Supplement: Supplementary file 1 [file nutrients-17-02729-s001.zip › Supplementary_Information_6.6.2025.pdf]

Targeted Microbial Shifts and Metabolite P rofiles Were Associated with Clinical Response to an Anti-Inflammatory Diet in Osteoarthritis

Marta Sala-Climent [1,†], Kevin Bu [2,†], Roxana Coras [1], Martha Cedeno [1], Simone Zuffa [3], Jessica Murillo-Saich [1], Helena Mannochio-Russo [3], Celeste Allaband [4], Michal K. Hose [5], Anna Quan [5], Soo-In Choi [1], Katherine Nguyen [1], Shahrokh Golshan [6], Rebecca B. Blank [7], Tiffany Holt [6], Nancy E. Lane [8], Rob Knight [4,9,10,11], Jose Scher [7], Pieter Dorrestein [3], Jose Clemente [2,*] and Monica Guma [1,5,*]

SUPPLEMENTARY INFORMATION

Study Participants and Study Design: Patients aged 18 or more with a diagnosis of knee OA (KOA) with a visual analogue pain knee score of between 20-80 during the last 7 days, and without changes in therapy during the previous 3 months, were recruited at Rheumatology Outpatient Clinic of the University California San Diego and VA San Diego Health Care. The study was approved by the local institutional review boards (IRB: H170083 and 161474) and the procedures followed were in accordance with the ethical standards of the responsible committee on human experimentation. All patients signed an informed consent. Patients who were pregnant or lactating, already following a vegetarian or vegan diet, known food allergies, or patients who needed a change in OA medication regimen during the length of the study (30 days (D)) were excluded. We did not change their previous treatments. Sixty patients were screened, thirty-four patients were recruited, and twenty-one patients went through the complete trial. **SupplementaryTableS1** shows feasibility outcomes of the trial.

A prospective, open label pilot trial was conducted to evaluate the feasibility and clinical and biological outcomes of a 4-week isocaloric ITIS diet. The study was performed from September 2021 to March 2023. Research visits were performed on 3 occasions during the study (Figure 1A). During their first visit (D-14), we established their clinical and biological baseline. On their second visit (D0), two weeks later, patients were given instructions on how to follow the energy-adjusted ITIS diet (**Supplementary Tables S2 and S3**) and were asked to complete a daily diet log for 2 weeks before starting the diet, followed by 4 weeks after starting the diet. On their third visit (D28), we

evaluated diet adherence, trial satisfaction, and clinical parameters. At each visit, pain and overall health was evaluated by the patient, using a Visual Analogue Scale (VAS) that ranged from 0 to 10. Western Ontario and McMaster Universities Arthritis Index (WOMAC), PROMIS sleep disturbance, the Physical Activity Scale for the Elderly (PASE), the Center for Epidemiological Studies Depression (CES-D) and the painDETECT questionnaires were collected in the three visits. Blood samples were also collected at each visit by research personnel into 10 ml BD Vacutainer Blood Collection Tubes containing EDTA. Tubes were centrifuged for 20 min at 2000×rpm and plasma were transferred into 1.7ml tubes and immediately frozen and stored at −80 °C until analysis. We collected blood at all visits. Patients were asked to collect stool samples at home after each visit with swabs, three for stool and one for saliva, provided on the day of the visit. Stool samples were shipped in containers, aliquoted and kept at -80 °C until analysis.

Adherence to Dietary Intervention and Diet Score: The subjects completed a Food Frequency Questionnaire (FFQ) on D-14. They also completed a daily record 2 weeks before and 4 weeks during the diet intervention, recording whether they had consumed the suggested meals and ingredients, which options they had chosen for breakfast and meals, and if there was any consumption of forbidden ingredients. We calculated a diet score **(Supplementary Table S4)** for the evaluation of patient adherence. Consumption of anti-inflammatory foods according to the proposed diet received a positive evaluation. Pro-inflammatory foods, i.e., red meat or Solanaceae vegetables received a negative evaluation. We considered this score as the gold standard and used it to characterize the patient's baseline diet as well.

Primary and Secondary Outcomes: Change in pain score was the primary outcome. According to Conaghan et al. we considered a 30% reduction in WOMAC pain to be an appropriate threshold for patients with OA.[1] Secondary outcomes included change of other clinical scores such as VAS, activity or sleep quality, gut and saliva microbiota, gut and plasma metabolome.

Statistical analysis of Outcomes: Continuous variables are presented as the mean ± standard deviation (SD), whereas categorical variables are summarized as the number (percentage) of subjects. Nonparametric tests were used to compare means across 2 groups. For the analysis of longitudinal data, paired Wilcoxon-test were used when samples matched across 2 timepoints were compared. Data were analyzed with R 4.2.1 (https://posit.co/download/rstudio-desktop/). The barplots were built using the *ggboxplot* function and the line plots using the *ggline* function in the *ggplot2 (3.5.1)* package. Microbiome and metabolomic data were analyzed using nonparametric

statistical tests and paired when appropriate. Differences in alpha diversity between responders and non-responders were assessed using Wilcoxon Rank-Sum, and differences in alpha diversity between pre- and post- intervention timepoints were tested using Wilcoxon Signed-Rank. Beta diversity was compared using PERMANOVA (with 1000 iterations). Univariate statistical testing for individual features was done using Wilcoxon Rank-Sum or Wilcoxon Signed-Rank and p-values were adjusted using FDR-BH. Correlation analyses were conducted using Spearman correlation (or Pearson when distributional assumptions were met) and confounders and covariates were controlled for using generalized linear models. All statistical tests were performed in Python 3.7.3 using the scipy.stats library and in R 4.2.1 using the base library. Filtered quantification table of microbiome, metabolome and the metadata (.csv) files was uploaded to the OmicsAnalyst 2.0[2] platform for Data Integration Analysis for Biomarker discovery using Latent cOmponents (DIABLO), a multi-omics method that simultaneously identifies key omics variables during the integration process and discriminates phenotypic groups.

Untargeted Metabolomics

Sample preparation: Plasma

Three hundred µL of MeOH (100%) was added to each well of the 96-well plate Phree Phospholipid Removal Kit and centrifuged at 500 g for 5 minutes, 3 times prior to sample addition; the MeOH was discarded in the laboratory hazardous waste. Blood plasma was stored at −80°C prior to extraction in 1.5 mL microtubes. The blood plasma microtubes were thawed at room temperature prior to extraction. Blood plasma samples were placed into one of four Phree Phospholipid Removal Kit 96-well plates randomly. The thawed blood plasma samples were vortexed for 5 seconds and centrifuged for 1 minute at 5,000 rpm prior to pipetting 50 µL of each sample into the 96-well Phree Phospholipid Removal Kit. Two hundred µL of MeOH (100%) was added to each well using a multichannel pipette; the solution was aspirated and dispensed 5 times to mix the blood plasma and organic solvent. A 96-well plate (Eppendorf Microplate 96/U-PP) was placed under the Phree Phospholipid Removal Kit to collect the sample and centrifuged at 500 g for 5 minutes. The Phree Phospholipid Removal Kit portion was discarded in the solid biohazardous waste and the sample-containing 96-well plate was evaporated until dry using a CentriVap Benchtop Vacuum Concentrator (Labconco, Kansas City, MO). The 96-well plate containing the dried extract was covered (Storage Mat III 3080) and stored at −80°C prior to

analysis. Immediately prior to analysis, the dried extract material was resuspended in 200 µL of MeOH-water (1:1), sonicated for 5 minutes, centrifuged for 5 minutes at 500 g, and covered with a plate-sealing film (Zone-Free Sealing Films).

Sample preparation: Feces

Fecal samples were stored at −80°C prior to extraction. The swab tip of the BD Falcon SWUBE Collection and Transport System swabs were cut into Nunc 96-Well Polypropylene DeepWell Storage Plates. Sample barcodes were scanned using a barcode scanner and saved into a Google Sheets spreadsheet generating a record of which sample was positioned in each well of the plate. Three hundred µL of MeOH-water (1:1) was added to each well using a multichannel pipette. The deep well plate was covered with a storage mat and floated in an ultrasonic bath for 5 minutes. The samples were placed in a 4°C refrigerator overnight to extract. Subsequently, the swabs were removed from each well using tweezers, rinsing in between with nanopure water. The swab tips were disposed of in the solid biohazardous waste. Extracts were evaporated until dry using a CentriVap Benchtop Vacuum Concentrator (Labconco, Kansas City, MO, USA). The 96-well plates containing the dried extract were covered (96-deep well plate mats, Nunc 96 Well Caps for 1.0 mL Polystyrene DeepWell Plates) and stored at −80°C prior to analysis. Immediately prior to analysis, the dried extract material was resuspended in 300 µL of MeOH-water (1:1), sonicated for 5 minutes, and centrifuged for 5 minutes at 500 g. One hundred µL of extract from each well was transferred into a 96-well plate (Eppendorf Microplate 96/U-PP) and diluted twofold using MeOH-water (1:1) and covered with a plate-sealing film (Zone-Free Sealing Films).

Data acquisition: Blood plasma and fecal samples were analyzed using LC-MS/MS data acquisition which was performed on a Vanquish ultrahigh-performance liquid chromatography (UPLC) system using a core-shell silica C18 column (2·1 x 50 mm, 1·7-µm particle size, 100-Å pore size; Kinetex, Phenomenex) coupled to a Q Exactive Orbitrap mass spectrometer (Thermo Fisher Scientific, Bremen, Germany). Five microliters of sample were injected and run at 0.5 ml/min on a gradient of solvent A (HPLC-grade water with 0·1% formic acid) and solvent B (HPLC-grade acetonitrile with 0.1% formic acid). The column was maintained at 40 °C. The UPLC elution gradient ran for 12·5 min per sample: 5% B from 0 min to 1 min, a linear gradient of 5 to 100% B over 8 min, a hold at 100% B for 2 min, a return to 5% B over 0·5 min, and a hold at 5% B for 2 min to equilibrate the column for the next sample. The flow was directed into a heated electrospray ionization source operated in positive ionization mode with the following

parameters: an auxiliary gas flow rate of 14 arbitrary units (a.u.), sweep gas flow rate of 3 a.u., sheath gas flow rate of 52 a.u., spray voltage of +3·5 kV, capillary temperature of 270°C, auxiliary gas heater temperature of 435°C, and S-Lens RF level of 50. The data-dependent acquisition mode was used to acquire the data in which MS1 scans from *m/z* 100 to 1,500 (scan rate, 7 Hz) were followed by an MS2 scan, specifically a product ion scan produced using stepped normalized collision energy higher-energy collisional dissociation, of the five most abundant ions from the prior MS1 scan.

Data processing and data analysis: Raw data were uploaded to MassIVE (https://massive.ucsd.edu/), converted to .mzML files, imported into MZmine2 [3], and truncated at *m/z* 1500 and a 9.5-min retention time (RT). The parameters used in MZmine2 are as follows. Mass detection was performed with a noise threshold of 2·0e5 for MS1 and 2·0e3 for MS2 in centroid mode, and chromatograms were built with a 0·05-min time span, 1·0e6 minimum height, and 10-ppm m/z tolerance. Chromatograms were deconvoluted with the baseline cutoff algorithm and a minimum peak height of 1·0e6, peak duration of 0·05 to 1·0 min, and baseline of 1.0e4. Isotope peak removal was performed with 15-ppm *m/z* tolerance, 0.3 min RT tolerance, and maximum charge of 4, and peaks were aligned with 10-ppm *m/z* tolerance, 75 weight *m/z* tolerance, and 0·4 min RT tolerance. Gap filling was also performed with 10% intensity tolerance, 15-ppm *m/z* tolerance, and 0·3 min RT tolerance. Peaks were also filtered to remove singletons found in only one sample. Both MS1 and MS2 feature tables were exported, and the "export for GNPS" feature was used to generate a .mgf file for GNPS. The signal intensities of the MS1 features were normalized (probabilistic quotient normalization) to the sulfamethoxazine internal standard.

Molecular networking (GNPS): A molecular network was created with the feature based molecular networking workflow (https://ccms-ucsd.github.io/GNPSDocumentation/featurebasedmolecularnetworking/) on GNPS for blood
pasma (plasma), saliva (saliva) and stool (stool)[4]. The data were filtered by removing all MS2 product ions within ± 17 *m/z* of the precursor *m/z*. MS2 spectra were window filtered by choosing only the top 6 fragment ions in the ± 50 *m/z* window throughout the spectrum. The precursor *m/z* tolerance was set to 0·02 *m/z* and a MS2 product ion m/z tolerance of 0·02 *m/z*. A network was then created where edges were filtered to have a cosine score above 0·7 and at least 4 matched peaks. Further, edges between two nodes were kept in the network if and only if each of the nodes appeared in each other's respective top 10 most similar nodes. Finally, the maximum size of a

molecular family (i.e., network component) was set to 100, and the lowest scoring edges were removed from molecular families until the molecular family size was below this threshold. The spectra in the network were then searched against GNPS spectral libraries. The library spectra were filtered in the same manner as the input data. All matches kept between network spectra and library spectra were required to have a score above 0·7 and at least 4 matched peaks. The annotations are level 2 or 3 according to the 2007 metabolomics standards initiative. A total of 15,600 spectra were detected in stool, 15,936 in saliva, and 5,404 in plasma. Among these, 183 nodes in stool, 184 in saliva, and 184 in plasma were successfully annotated. After filtering out features with zero intensity across all samples, 88 annotations were retained in stool, 48 in saliva, and 28 in plasma.

Metabolomics data availability: All MS data (.d and .mzXML files) are publically available via GNPS/MassIVE (massive.ucsd.edu), a public MS data repository, under the accession number **MSV000094097**. Differentially abundant features (metabolites) between pain 50 improvement and study D+14 and -14 were calculated through Songbird (see Differential abundance and multi-omics analyses). Posteriorly, only the annotated metabolites were used to generate the barplots presented in the figures. The comparisons for all the features are presented as supplementary tables.

Microbiome

Sample and data processing: 20 patients provided fecal samples, which were sequenced The UC San Diego Microbiome Core performed nucleic acid extractions utilizing previously published protocols.[40] The process of microbiome sample purification was carried out using the MagMAX Microbiome Ultra Nucleic Acid Isolation Kit (Thermo Fisher Scientific, USA). This kit is designed to effectively isolate nucleic acids from a variety of sample types, ensuring the removal of contaminants and preserving the integrity of the microbial DNA. The purification steps were automated using KingFisher Flex robots (Thermo Fisher Scientific, USA), which are equipped with specialized protocols for high-throughput sample processing, ensuring consistent and efficient purification across multiple samples simultaneously. To quantify the extracted DNA, a PicoGreen fluorescence assay (Thermo Fisher Scientific, USA) was used. This assay is sensitive and accurate, allowing for the measurement of low concentrations of DNA by detecting fluorescence emitted by the PicoGreen reagent when bound to double-stranded DNA. The results were used to determine the DNA concentrations of the samples, ensuring optimal amounts for

subsequent analyses. For the bacterial 16S rRNA gene sequencing, which targeted the V4 region of the gene, Illumina MiSeq technology was employed. This sequencing platform is known for its high-throughput capabilities and is suitable for amplifying and sequencing microbial communities in environmental and clinical samples. The V4 region of the 16S rRNA gene was amplified using specific primers and sequenced with paired-end 150 bp cycles, which provide a detailed view of microbial diversity. This sequencing approach is widely used in microbiome studies due to its ability to capture a broad spectrum of bacterial taxa.

Microbiome Data Processing Raw 16S sequencing data was analyzed using Quantitative Insights Into Microbial Ecology (Qiime2-2020.8.0)[5]. Sequences were demultiplexed, denoised using DADA2[6], and mapped to ASVs using the Greengenes 13_8 database[7]. The resulting feature table was rareified to 5,000 reads per sample before performing alpha-diversity (Shannon Entropy) and beta-diversity (unweighted UniFrac) calculations in Qiime2. Alpha-diversity statistical significance was assessed using either Wilcoxon Rank-Sum or Signed-Rank for unpaired and paired samples respectively. Beta-diversity statistical significance between sample groups was assessed through permutational multivariate analysis of variance (PERMANOVA). Univariate differential abundance analyses were conducted on the normalized abundances using Wilcoxon Signed-Rank and Rank-Sum tests, while multivariate differential analyses models were conducted using MaAsLin2 using default parameters[8]. Correlation analyses were performed using Pearson's correlation coefficient when assumptions of normality were not violated, and otherwise Spearman's Correlation was used. For paired data, correlations were conducted on the differences in values (post minus pre) while for unpaired data, all samples were used. Statistical tests were performed using the base stats library in R (4.4.1), while barplots, boxplots, scatterplots and other visualizations were generated using ggplot2 and ggpubr.

Supplementary Table S1. Feasibility outcomes of the trial. The table includes feasibility outcomes of the trial.

Supplementary Table S 2. Summary of Dietary Recommendations. The table includes recommendations of foods recommended to increase intake and foods which are recommended to decrease intake.

Supplementary Table S3. Proposed meal organization for the 4 weeks of the intervention.

Supplementary Table S4. Diet score calculation. The table shows the importance given to each type of food/ingredient in the calculation of the score. Positive scores were given to foods considered anti-inflammatory, while negative scores were assigned to foods considered pro-inflammatory.

Supplementary Table S5. Clinical outcomes on D-14 and D 0. The average values along with the standard deviations are presented, along with the significance (p values) of the comparison of the outcomes at the 2 timepoints (t-test).

Supplementary Table S6. **Change in diet scores after diet.**

Supplementary Table S7. **Outcomes differences between responders and no responders at baseline**

Supplementary Table S8. Changes in diet scores after diet by response to Womac Pain. The average values along with the standard deviations are presented, along with the significance (p values) of the comparison of the two groups.

Supplementary Table S 9. Change in diet scores differences between responders and no responders at baseline and after diet. The average values along with the standard deviations are presented, along with the significance (p values) of the comparison of the two groups.

Supplementary Table S10. Generalized Linear Models. Linear models relating the *change* in WOMAC_Pain to the *change* (post minus pre) in specified features. Gaussian noise assumption. No patients had a comorbidity of diabetes.

REFERENCES

1. Conaghan PG, Dworkin RH, Schnitzer TJ, et al. WOMAC Meaningful Within-patient Change: Results From 3 Studies of Tanezumab in Patients With Moderate-to-severe Osteoarthritis of the Hip or Knee. J Rheumatol 2022;49:615-621.

2. Ewald JD, Zhou G, Lu Y, et al. Web-based multi-omics integration using the Analyst software suite. Nat Protoc 2024;19:1467-1497.

3. Kessner D, Chambers M, Burke R, Agus D, Mallick P. ProteoWizard: open source software for rapid proteomics tools development. Bioinformatics. 2008 Nov 1;24(21):2534-6. doi: 10.1093/bioinformatics/btn323. Epub 2008 Jul 7. PMID: 18606607; PMCID: PMC2732273.

4. Wang M, Carver JJ, Phelan VV, Sanchez LM, Garg N, Peng Y, et al . Sharing and community curation of mass spectrometry data with Global Natural Products Social Molecular Networking. Nat Biotechnol. 2016 Aug 9;34(8):828-837. doi: 10.1038/nbt.3597. PMID: 27504778; PMCID: PMC5321674.

5. Bolyen E, Rideout JR, Dillon MR, Bokulich NA, Abnet CC, Al-Ghalith GA, et al . Reproducible, interactive, scalable and extensible microbiome data science using QIIME 2. Nature Biotechnology 37: 852–857. https://doi.org/10.1038/s41587-019-0209-9

6. Callahan BJ, McMurdie PJ, Rosen MJ, Han AW, Johnson AJ, Holmes SP. DADA2: High-resolution sample inference from Illumina amplicon data. Nat Methods. 2016 Jul;13(7):581-3. doi: 10.1038/nmeth.3869. Epub 2016 May 23. PMID: 27214047; PMCID: PMC4927377.

7. McDonald D, Jiang Y, Balaban M, Cantrell K, Zhu Q, Gonzalez A, et al. Greengenes2 unifies microbial data in a single reference tree. Nat Biotechnol. 2024 May;42(5):715-718. doi: 10.1038/s41587-023-01845-1. Epub 2023 Jul 27. Erratum in: Nat Biotechnol. 2024 May;42(5):813. doi: 10.1038/s41587-023-02026-w. PMID: 37500913; PMCID: PMC10818020.

8. Mallick H, Rahnavard A, McIver LJ, Ma S, Zhang Y, Nguyen LH, Tickle TL, Weingart G, Ren B, Schwager EH, Chatterjee S, Thompson KN, Wilkinson JE, Subramanian A, Lu Y, Waldron L, Paulson JN, Franzosa EA, Bravo HC, Huttenhower C (2021). [Multivariable Association Discovery in Population-scale Meta-omics Studies]
