# Supplementary figures and images for: Targeted Microbial Shifts and Metabolite Profiles Were Associated with Clinical Response to an Anti-Inflammatory Diet in Osteoarthritis
Source: Nutrients. 2025 Aug 22;17(17):2729. doi: 10.3390/nu17172729 (PMC12430150; doi:10.3390/nu17172729)

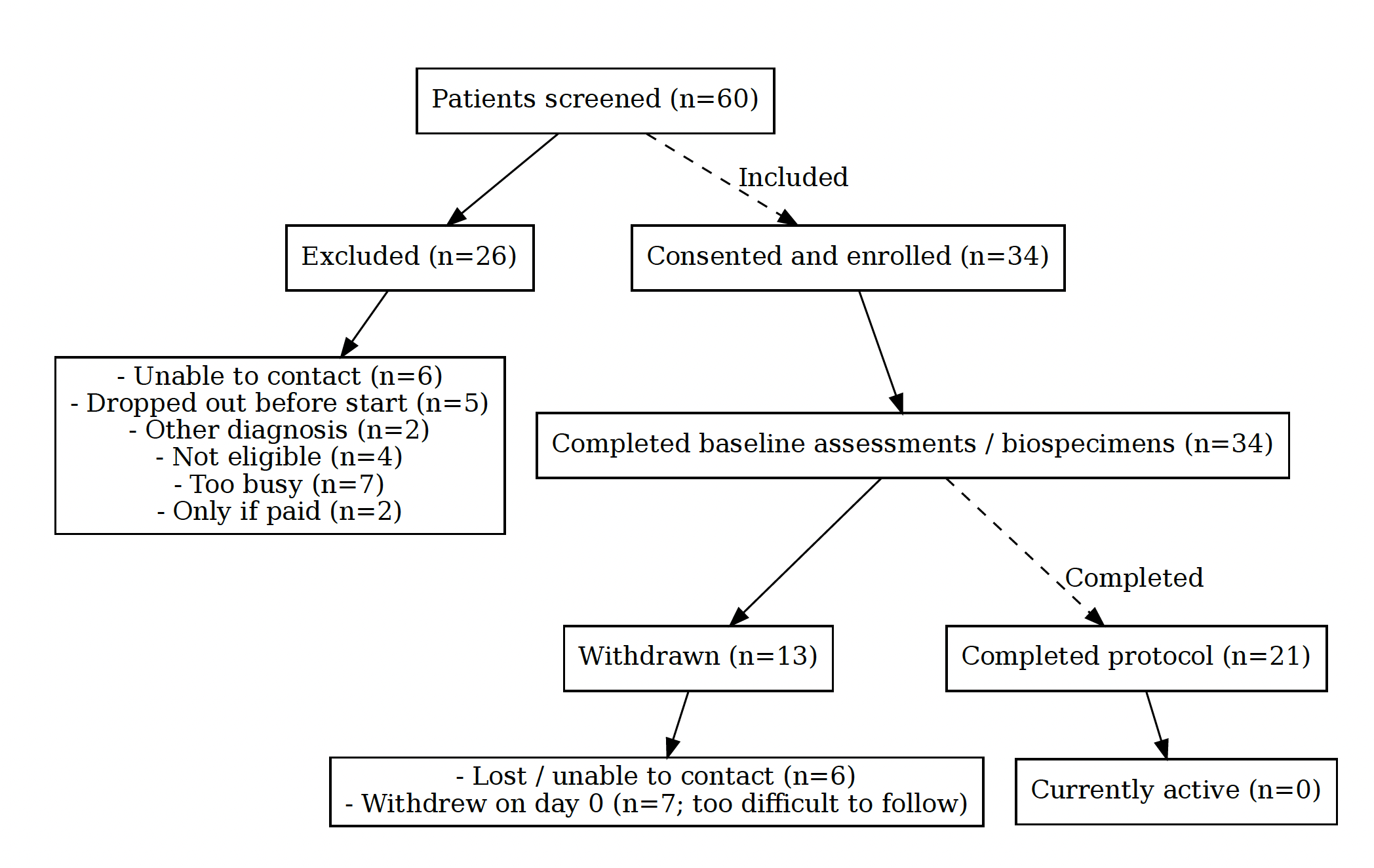

Supplement: Supplementary file 1 [file nutrients-17-02729-s001.zip › Supplementary Figures/OA_SF_1.png]

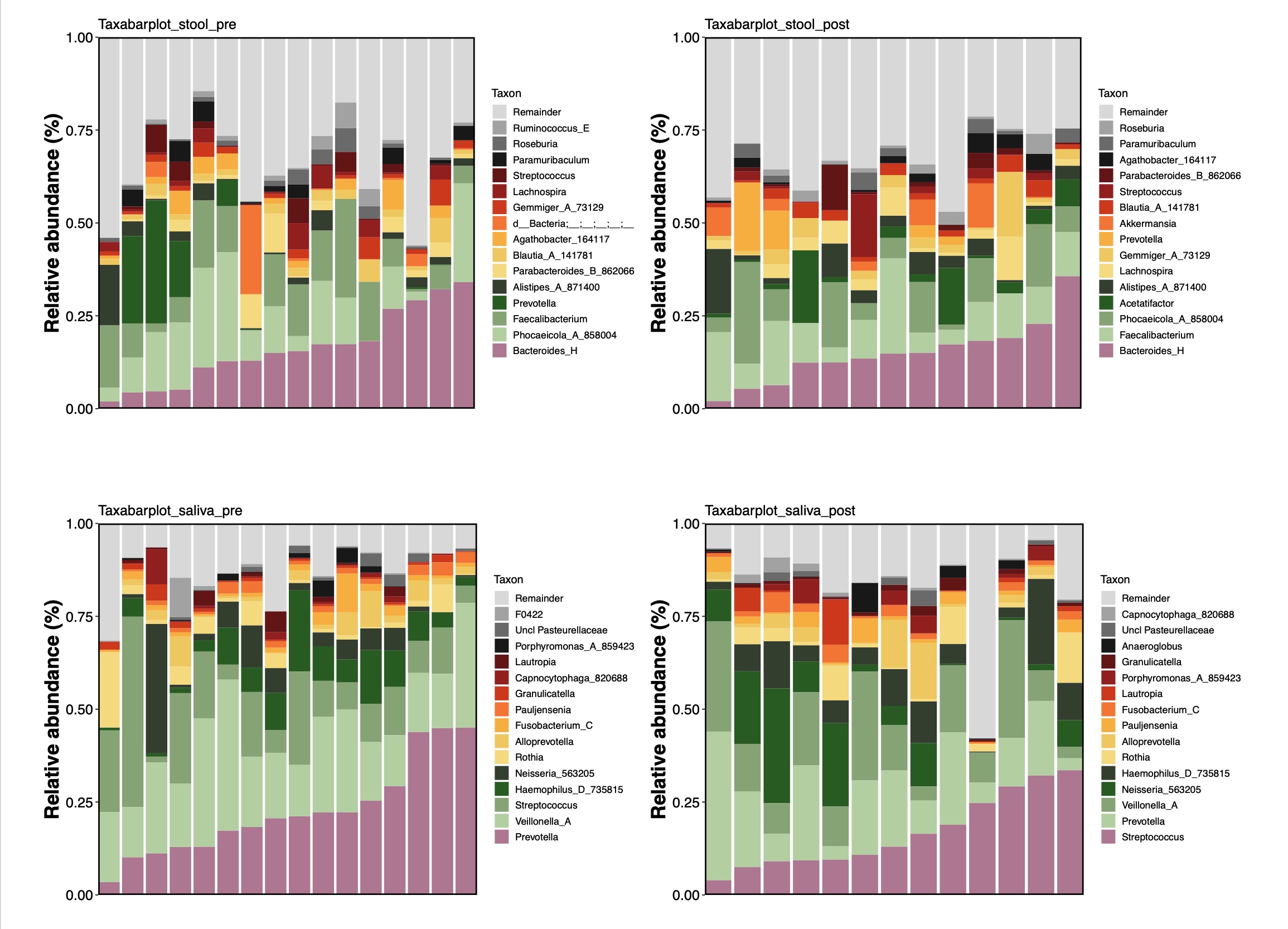

Supplement: Supplementary file 1 [file nutrients-17-02729-s001.zip › Supplementary Figures/OA_SF_2.jpg]

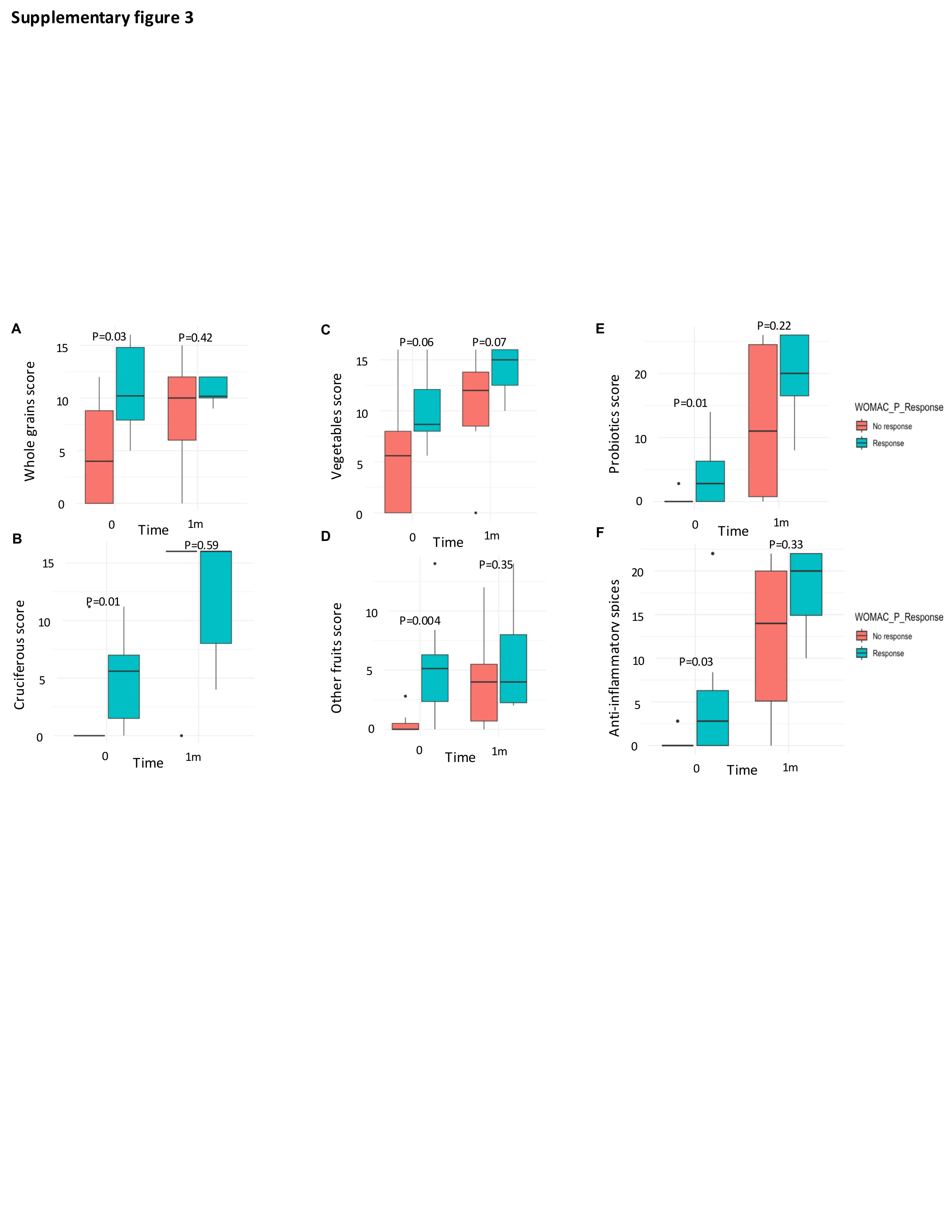

Supplement: Supplementary file 1 [file nutrients-17-02729-s001.zip › Supplementary Figures/OA_SF_3.jpg]

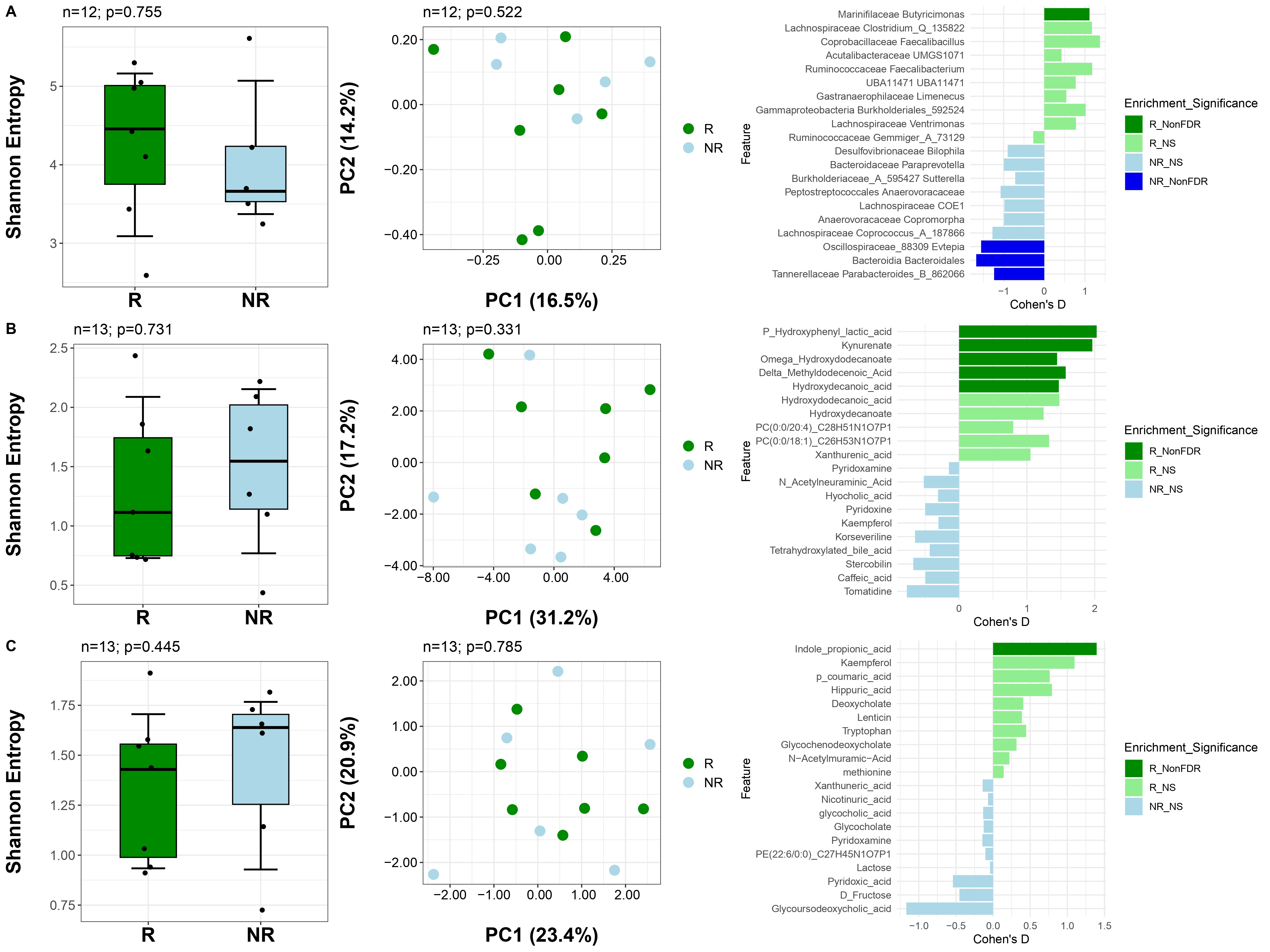

Supplement: Supplementary file 1 [file nutrients-17-02729-s001.zip › Supplementary Figures/OA_SF_4.jpg]

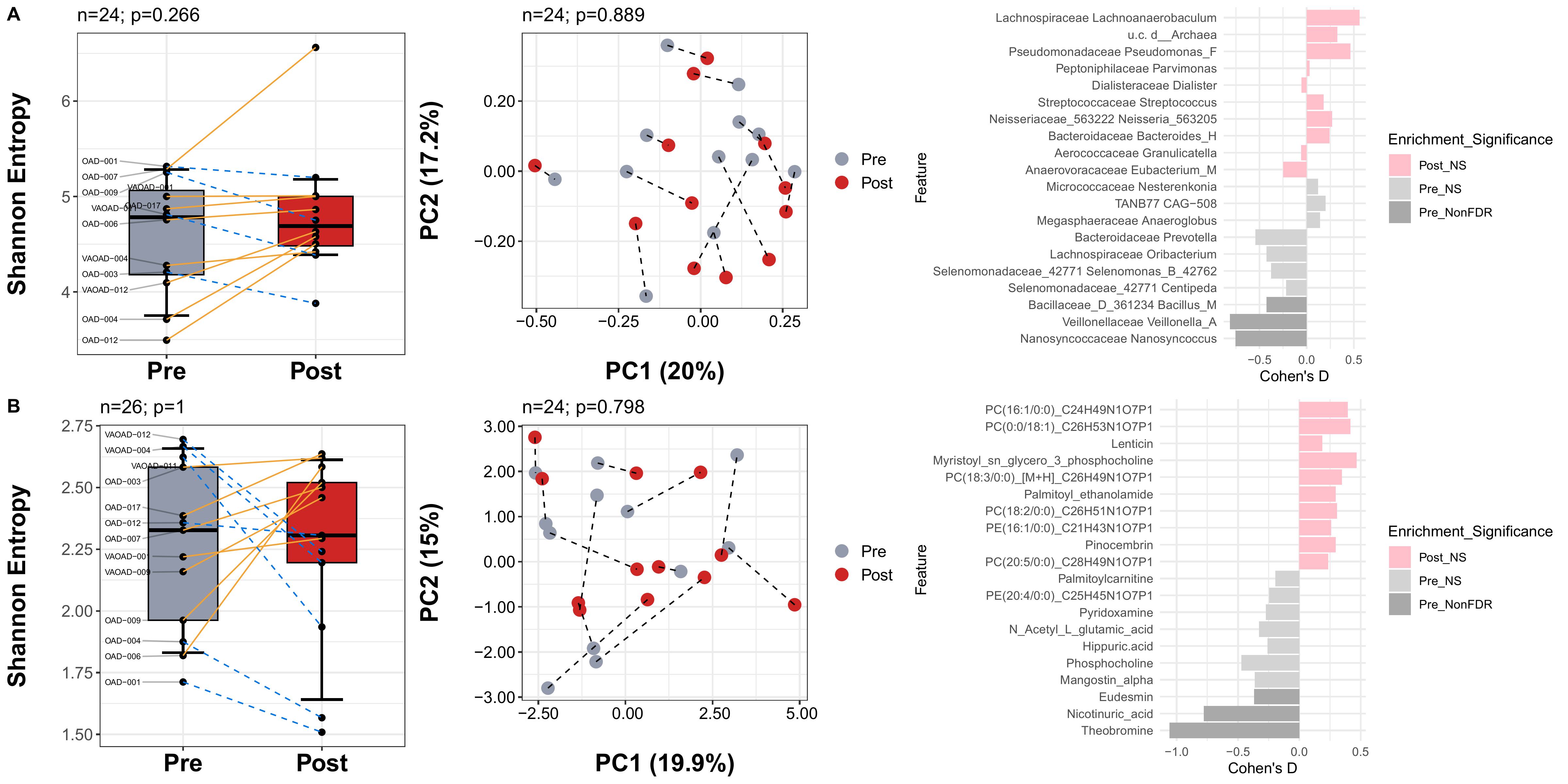

Supplement: Supplementary file 1 [file nutrients-17-02729-s001.zip › Supplementary Figures/OA_SF_5.jpg]

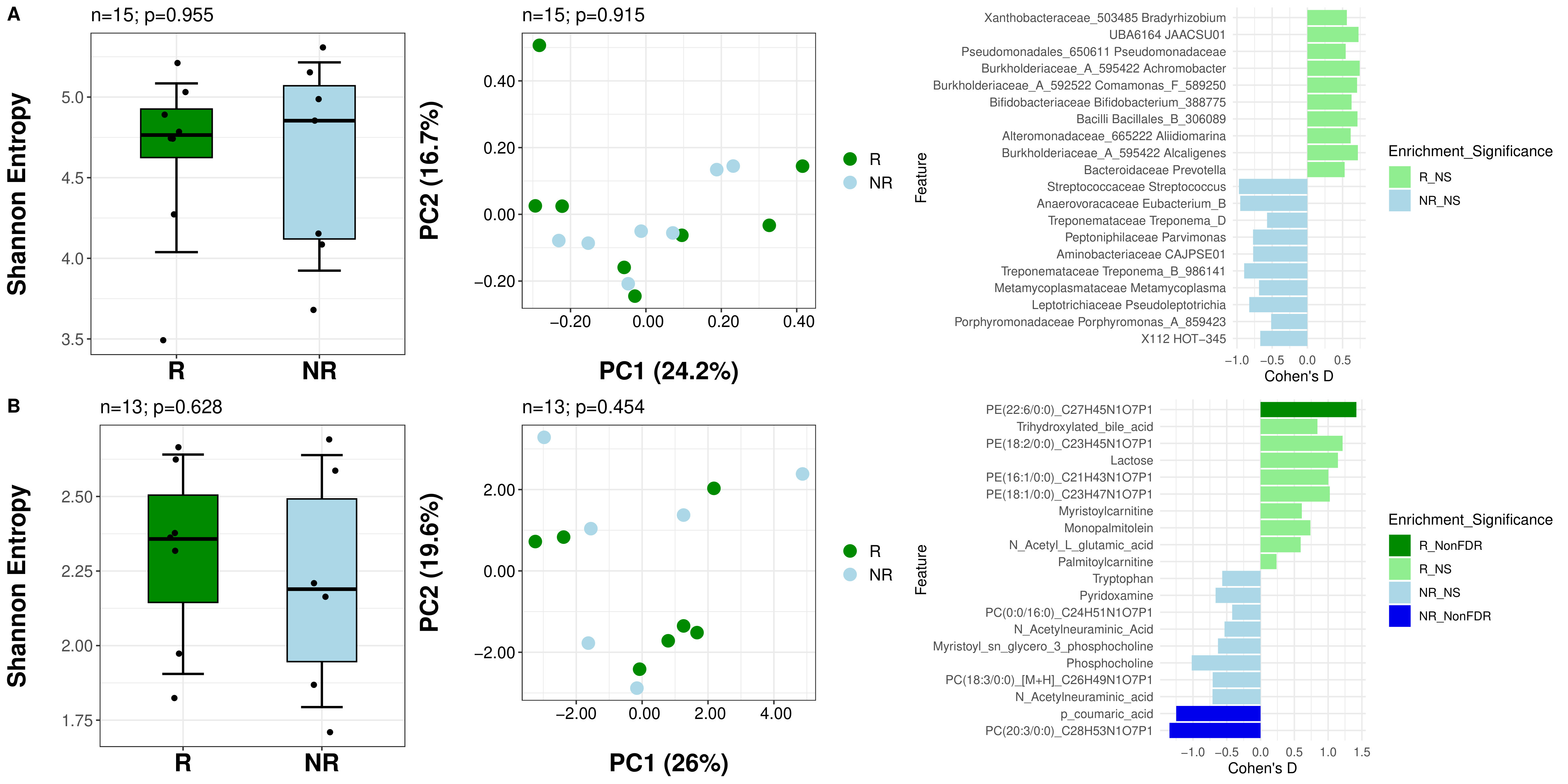

Supplement: Supplementary file 1 [file nutrients-17-02729-s001.zip › Supplementary Figures/OA_SF_6.jpg]

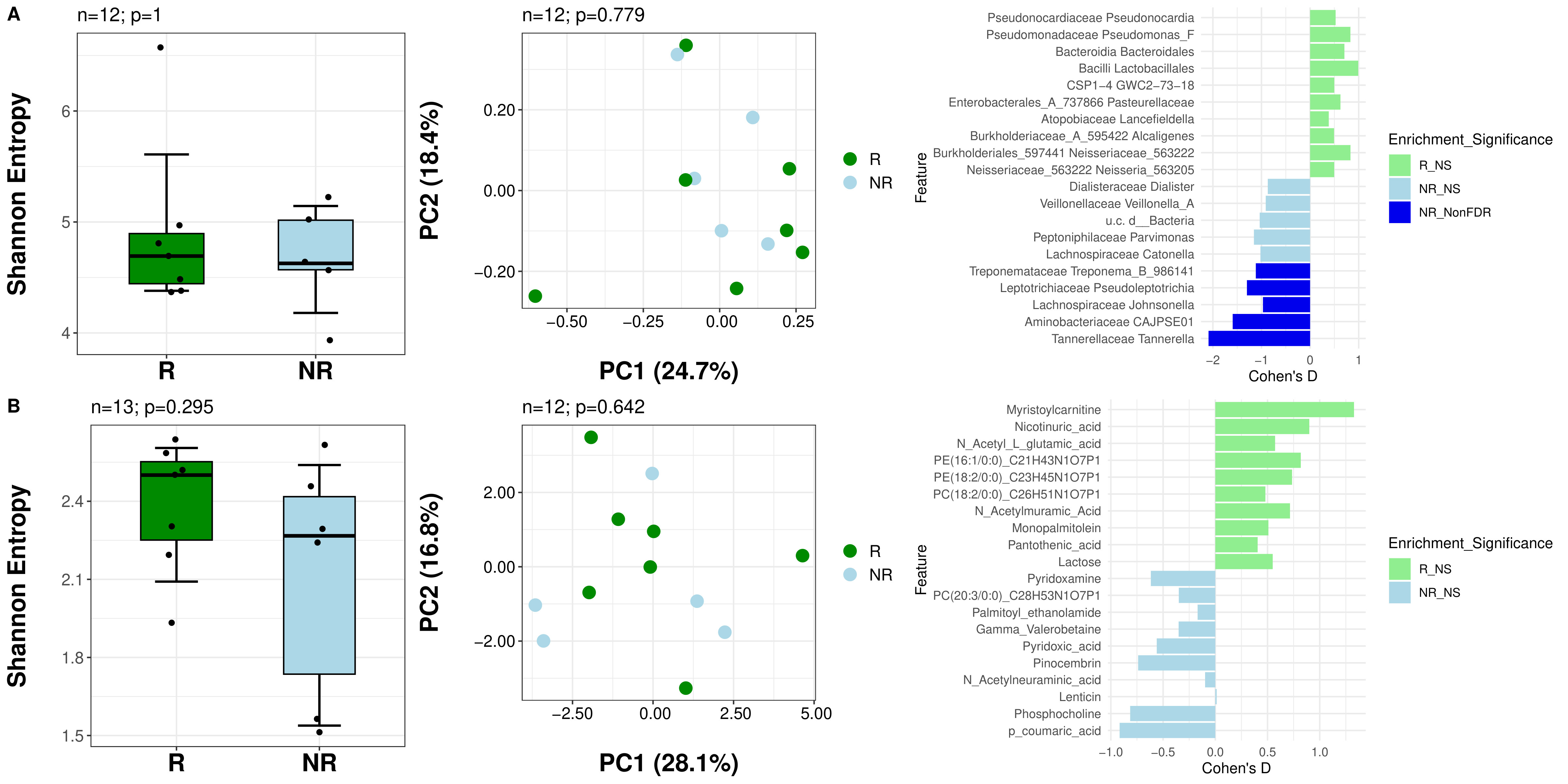

Supplement: Supplementary file 1 [file nutrients-17-02729-s001.zip › Supplementary Figures/OA_SF_7.jpg]

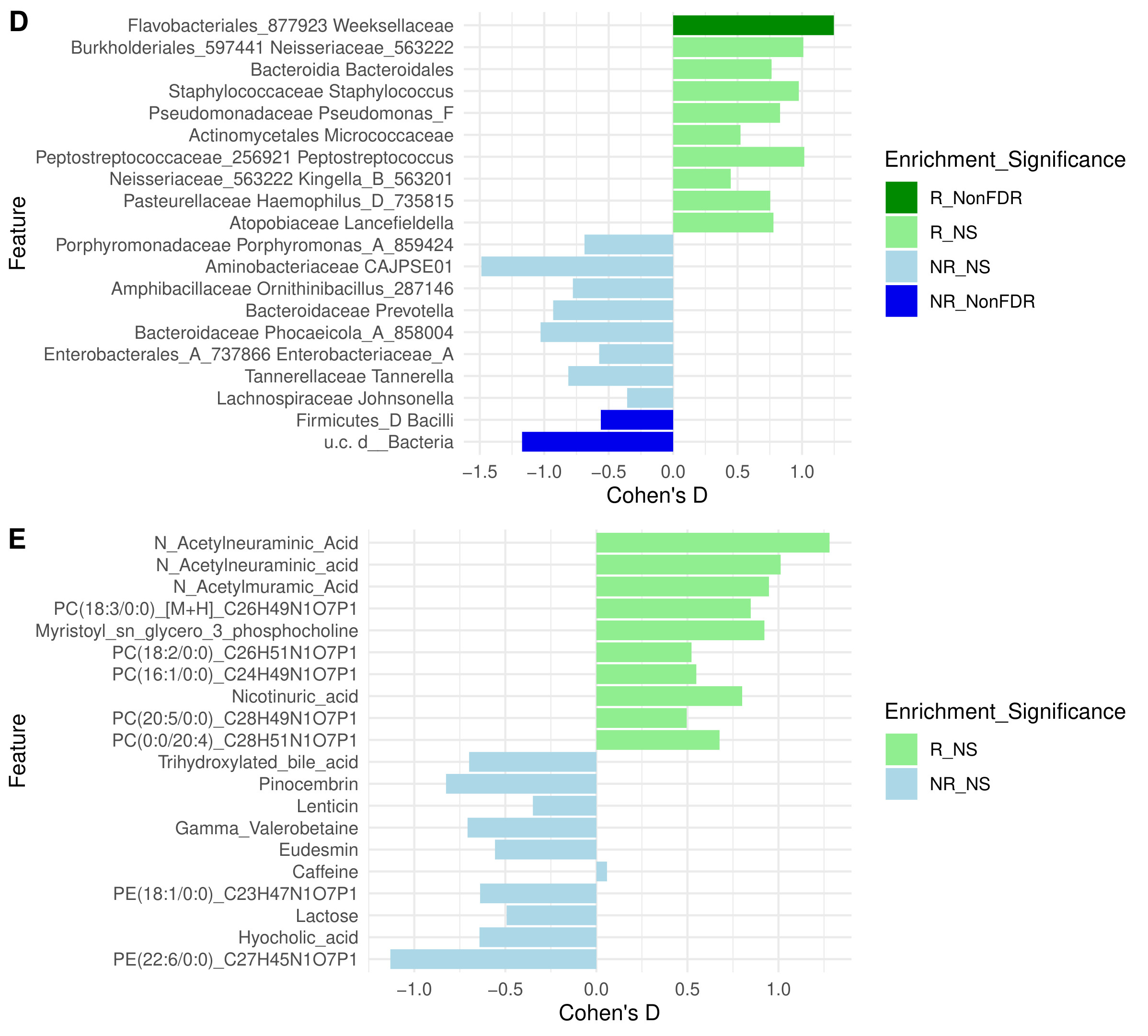

Supplement: Supplementary file 1 [file nutrients-17-02729-s001.zip › Supplementary Figures/OA_SF_8.jpg]
